# Influenza A(H5N1) Virus Clade 2.3.2.1a in Traveler Returning to Australia from India, 2024

**DOI:** 10.3201/eid3101.241210

**Published:** 2025-01

**Authors:** Yi-Mo Deng, Michelle Wille, Clyde Dapat, Ruopeng Xie, Olivia Lay, Heidi Peck, Andrew J. Daley, Vijaykrishna Dhanasakeran, Ian G. Barr

**Affiliations:** World Health Organization Collaborating Centre for Reference and Research on Influenza, Peter Doherty Institute for Infection and Immunity, Melbourne, Victoria, Australia (Y.-M. Deng, M. Wille, C. Dapat, O. Lay, H. Peck, I.G. Barr); The University of Melbourne, Melbourne (M. Wille, I.G. Barr); The University of Hong Kong, Hong Kong, China (R. Xie, V. Dhanasakeran); The Royal Children's and Royal Women's Hospitals, Parkville, Victoria, Australia (A.J. Daley)

**Keywords:** influenza, viruses, avian influenza, H5N1, highly pathogenic, human, traveler, Australia, India, respiratory infections

## Abstract

We report highly pathogenic avian influenza A(H5N1) virus clade 2.3.2.1a in a child traveler returning to Australia from India. The virus was a previously unreported reassortant consisting of clade 2.3.2.1a, 2.3.4.4b, and wild bird low pathogenicity avian influenza gene segments. These findings highlight surveillance gaps in South Asia.

The global panzootic of highly pathogenic avian influenza (HPAI) A(H5N1) clade 2.3.4.4b is affecting wild and domestic birds and mammals worldwide (*1*). This viral clade emerged after decades of evolution of goose/Guangdong (gs/Gd) lineage HPAI H5N1 viruses, first detected in geese in China in 1996 (*2*). Although clade 2.3.4.4b is globally dominant, a diversity of HPAI H5N1 clades are present in poultry in Asia today. Since 2005, >900 zoonotic infections have been recorded, primarily caused by contact with infected poultry (*3*); no evidence exists of human-to-human transmission. Reflecting the diversity of gs/Gd clades in Asia, human infections in Asia have been caused by a variety of clades. For example, Cambodia recorded 11 human infections caused by HPAI H5N1 clade 2.3.2.1c in the past 2 years, and China recorded 91 human infections caused by HPAI H5N6 and 2 cases caused by clade 2.3.4.4b H5N1 since 2014, many of them fatal.

Clade 2.3.2.1a continues to be detected in South Asia, particularly in India and Bangladesh. However, human infections have been rare; to our knowledge, only 2 cases have been reported (*4*,*5*). In recent years, several poultry outbreaks of H5N1 have occurred in India (*6*), raising concerns about the spread and evolution of clade 2.3.2.1a HPAI H5N1 viruses. We describe HPAI H5N1 clade 2.3.2.1a infection in a traveler returning to Australia from India.

## The Study

A previously healthy 2.5-year-old girl returned to Melbourne, Victoria, Australia, after visiting Kolkata, India, during February 12–29, 2024. The child became ill in India; her family sought medical care on February 28. After returning to Australia, she was hospitalized on March 2, then transferred with severe influenza on March 4 and admitted to intensive care with respiratory failure requiring mechanical ventilation. Influenza A virus was detected by PCR, but no subtyping was performed. A 5-day course of oseltamivir was administered beginning on day 3 after admission; she recovered fully and was discharged after 2.5 weeks. No clinical illness was apparent in other family members, and no samples were taken or tested (*7*,*8*).

A nasopharyngeal swab sample taken 2 days after admission and an endotracheal aspirate sample taken 3 days after admission were sent to the World Health Organization Collaborating Centre for Reference and Research on Influenza in Melbourne, followed by other routine influenza samples a month later. We identified H5N1 virus, designated as A/Victoria/149/2024(H5N1) (GISAID accession no. EPI_ISL_19156871; https://www.gisaid.org), by routine next-generation sequencing. In brief, we amplified influenza A genomes with Uni12/Inf-1 and Uni13/Inf-3 primers (*9*). We used PCR amplicons for Nanopore library preparation with rapid ONT barcoding kit (Oxford Nanopore Technologies, https://nanoporetech.com), loaded them into a standard flow cell, and sequenced them (MinION Mk1b for 8 hours). We analyzed sequence data using the IRMA pipeline (*10*). A multiple basic amino acid cleavage site motif in the hemagglutinin (HA) gene (PQKERRRKR*G) indicated the virus was an HPAI. 

**Figure 1:**
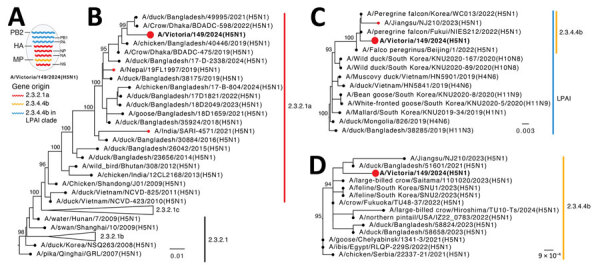
Evolutionary origins of isolate A/Victoria/149/2024(H5N1) (red circles and bold text) in study of influenza A(H5N1) virus clade 2.3.2.1a in traveler returning to Australia from India, 2024. A) Reassortant origins of A/Victoria/149/2024 based on analysis of each segment; detailed phylogenies for all segments are provided in Appendix Figure 1). B–D) Maximum-likelihood trees for HA (B), PB2 (C), and M (D) genes with a sample of BLAST-matched sequences (https://blast.ncbi.nlm.nih.gov). Bootstrap values >90% for key nodes are shown. Scale bars indicate number of nucleotide substitutions per site for each gene. HA, hemagglutinin; LPAI, low pathogenicity avian influenza; M, matrix protein; NP, nucleoprotein; NS, nonstructural; PA, polymerase acidic; PB1, polymerase basic 1; PB2, polymerase basic 2.

Phylogenetic analysis showed A/Victoria/149/2024(H5N1) was a reassortant virus (Figure 1; Appendix Figure 1). Four segments (HA, neuraminidase [NA], nucleoprotein, and nonstructural) were most similar to clade 2.3.2.1a viruses circulating in Bangladesh (Appendix Table). Two human cases of clade 2.3.2.1 were previously reported, 1 in India in 2021 (A/India/SARI14571) (*4*) and 1 in Nepal in 2019 (A/Nepal/19FL1997/2019) (*5*); those isolates shared basal lineages in their HA genes, indicating a common source. Further analysis of the A/Victoria/149/2024 HA sequence on a maximum-clade credibility tree demonstrated that the virus diverged from the most closely related clade 2.3.2.1a HA sequences in GISAID in June 2020 (95% highest posterior density December 2019–January 2021) (Appendix Figure 2). The matrix segment was similar to HPAI H5N1 clade 2.3.4.4b viruses, which continue to cause a global panzootic and have been detected in birds in Asia. The polymerase basic 2 (PB2), polymerase basic 1 (PB1), and polymerase acidic (PA) segments clustered with recent clade 2.3.4.4b low pathogenicity avian influenza viruses detected in wild birds and poultry in Asia since 2020, suggesting that clade 2.3.4.4b viruses likely served as intermediaries in transferring low pathogenicity avian influenza internal genes to the clade 2.3.2.1a virus we report, a genotype previously unreported in poultry or humans in South Asia. A few minor variants or polymorphisms were detected in PB2 and PB1 genes, which had no known associations (PB2, C196Y [18%], V344M [11%], M631L [16%]; PB1, L598P [16%]).

**Figure 2:**
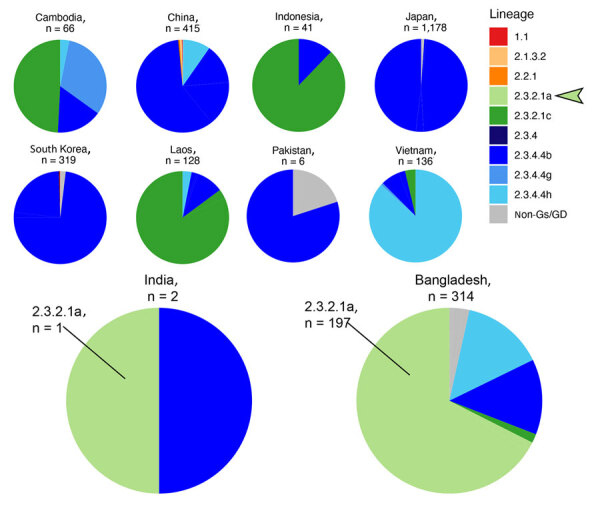
Numbers and diversity of highly pathogenic avian influenza A(H5Nx) virus isolates reported in South and East Asia in GISAID (https://www.gisaid.org) during January 1, 2020–July 29, 2024. Pie charts for India and Bangladesh, the only 2 countries in which clade 2.3.2.1a hemagglutinin sequences (light green, arrow) have been deposited into GISAID, have been enlarged.

During 2020–2024, only 322 H5 isolates were reported from South Asia (India, Pakistan, and Bangladesh) in GISAID; most arose from Bangladesh (n = 314) (Figure 2). Limited H5 data from India (2 sequences), combined with lack of information on patient exposure, makes contextualizing and determining whether the virus identified in this patient is representative of circulating H5 viruses in South Asia challenging.

We used FluSurver (https://gisaid.org/database-features/flusurver-mutations-app) to analyze the HA, PA, and NA segments for mammalian adaptation, virulence, and antiviral susceptibility. The HA sequence of the 220-loop receptor-binding site retained avian-like amino acids Q222 and G224, indicating retention of preferential binding to avian α2–3, and not the mammalian α2–6, sialic acid receptors. The virus contained no markers for mammalian adaptation or pathogenicity; avian-like E627, PB2, and D701 amino acids were retained, indicating no host adaptation or additional virulence markers (*11*). Those results mirror the features of the 2021 H5N1 human case in India (Figure 1, panel A) (*4*), including the multiple basic amino acid cleavage site with only 1 change (HA R323K); both cases lacked classic mammalian adaptations. The NA was most closely related to A/chicken/Bangladesh/18-B-569/2022 using BLAST (https://blast.ncbi.nlm.nih.gov) of the GISAID database, with 98% identity (Appendix Table). Interrogation of the NA and PA sequences indicated the virus would be susceptible to oseltamivir and baloxavir marboxil.

## Conclusions

This report of a human HPAI H5N1 case in a traveler returning from India highlights several issues. First, clinicians should be vigilant for serious influenza A cases in returned travelers from regions with circulating avian influenza viruses; subtyping is essential for such cases of influenza A to eliminate nonseasonal influenza infections. This step is crucial for early antiviral treatment, especially for the H5N1 and H5N6 viruses currently circulating in South/Southeast Asia, which can be serious or even fatal. 

Second, although global attention is focused on the panzootic clade 2.3.4.4b viruses, a relatively small number of human infections (<100) have been recorded, and few have been serious. This contrasts with ≈100 human cases of clade 2.3.4.4 H5N6 viruses in China and clade 2.3.2.1c H5N1 in Cambodia, which caused many deaths. 

Third, this case highlights the lack of H5N1 data from India. Clade 2.3.2.1a human infections in India and Nepal coincided with circulation in poultry and wild birds in Bangladesh (*12*). The fatal case in New Delhi in 2021, involving an 11-year-old boy who had contact with poultry (although no infected birds were reported) (*4*), is consistent with the genome reported here and genetically similar to H5N1 viruses present in Bangladesh. However, the patient in this study had no confirmed contact with poultry or raw poultry products; hence, the mode and route of infection cannot be determined. However, H5N1 was reported in 2023 and 2024 in Ranchi, India, 400 km from Kolkata (*13*,*14*). Since 2020, only 2 H5N1 sequences from India have been reported, compared with 314 H5N1 sequences from Bangladesh (197 in clade 2.3.2.1a) (Figure 2). Furthermore, the most recent common ancestor to A/Victoria/149/2024(H5N1) occurred almost 4 years before, highlighting the need for more sequence data from this region (Appendix Figure 2).

The complex reassortment origins of A/Victoria/149/2024(H5N1) show that clade 2.3.4.4.b viruses disseminated globally through wild birds might be transforming the genetic structure of other H5N1 clades endemic in poultry. Although HPAI H5N1 clade 2.3.4.4b viruses continue to be the focus of global attention, persistent HPAI H5Nx infections in Asia should not be overlooked.

Appendix Additional information about influenza A(H5N1) virus clade 2.3.2.1a in traveler returning to Australia from India, 2024.
