# Supplementary material for: Influenza A(H5N1) Virus Clade 2.3.2.1a in Traveler Returning to Australia from India, 2024
Source: Emerg Infect Dis. 2025 Jan;31(1):135–8. doi: 10.3201/eid3101.241210 (PMC11682823; doi:10.3201/eid3101.241210)
Supplement: Appendix — Additional information about influenza A(H5N1) virus clade 2.3.2.1a in traveler returning to Australia from India, 2024. [file 24-1210-Techapp-s1.pdf]

Article DOI: https://doi.org/10.3201/eid3101.241210

EID cannot ensure accessibility for supplementary materials supplied by authors. Readers who have difficulty accessing supplementary content should contact the authors for assistance.

Influenza A(H5N1) Virus Clade 2.3.2.1a in Traveler Returning to Australia from India, 2024

Appendix

Methods

The time-structured phylogenetic tree was estimated in BEAST v.1.10.4 (Appendix Figure 2) (*1*). The ML tree was used to generate a regression of root-to-tip genetic distances in TempEst (*2*), resulting high correlation coefficient of 0.83 and R^2 value of 0.69, indicating a strong temporal signal. BEAST analysis was performed using an SRD06 codon model, an uncorrelated lognormal relaxed molecular clock model, and a Bayesian Skyline tree prior. Three independent runs of 100 million generations were performed. Convergence was assessed using Tracer v1.6. Tree files were combined using LogCombiner v1.8 following removal of a 10% burn-in and Maximum clade credibility (MCC) trees were generated using TreeAnnotator v1.8. Trees were visualized with ggtree 3.10.1.

Results

The mean date of when A/Victoria/149/2024 diverged from the most closely related 2.3.2.1a HA sequences in GISAID was June 2020 (95% highest posterior density [HPD]: December 2019 - January 2021). That the most recent common ancestor (MRCA) was almost 4 years before the human case reported here underscores substantial missing or unsampled data, highlighting the need for more sequence data from the region.

References

1. Drummond AJ, Suchard MA, Xie D, Rambaut A. Bayesian phylogenetics with BEAUti and the BEAST 1.7. Mol Biol Evol. 2012;29:1969–73. PubMed https://doi.org/10.1093/molbev/mss075

2. Rambaut A, Lam TT, Max Carvalho L, Pybus OG. Exploring the temporal structure of heterochronous sequences using TempEst (formerly Path-O-Gen). Virus Evol. 2016;2:vew007. PubMed https://doi.org/10.1093/ve/vew007

Appendix Table. Top matches to each segment using BLAST in GISAID and Genbank (BLAST analysis performed on 15 July 2024)*

Segment	Database	Closest match	Clade	% identity
PB2	GISAID EpiFlu	EPI2752307 A/duck/Wakayama/22A6T/2022(H5N1)	2.3.4.4b	99%
	NCBI nt	LC774734 A/peregrine falcon/Fukui/NIES212/2022(H5N1)	2.3.4.4b	99.34%
PB1	GISAID EpiFlu	EPI2789452 A/environment/Kagoshima/KU-H1/2022 (H5N1)	2.3.4.4b	99%
	NCBI nt	OR950546 A/duck/Yunlin/20WB0265–1/2020(H1N6)	LPAI	98.33%
PA	GISAID EpiFlu	EPI2742639 A/white-fronted goose/Korea/22WC099/2022 (H5N1)	2.3.4.4b	99%
	NCBI nt	OR864262 A/duck/People’s Republic of China/JX02PA/2023(H4N6)	LPAI	98.75%
HA	GISAID EpiFlu	EPI2147679 A/duck/Bangladesh/46162/2020(H5N1)	2.3.2.1a	98%
	NCBI nt	OM938222 A/duck/Bangladesh/46157/2020(H5N1)	2.3.2.1a	98.53%
NP	GISAID EpiFlu	EPI2162602 A/chicken/Bangladesh/50592/2021(H5N1)	2.3.2.1a	99%
	NCBI nt	OP023558 A/chicken/Bangladesh/50585/2021(H5N1)	2.3.2.1a	99.13%
NA	GISAID EpiFlu	EPI3116264 A/chicken/Bangladesh/18-B-569/2022(H5N1)	2.3.2.1a	98%
	NCBI nt	PP680409 A/duck/Bangladesh/58667/2023(H5N1)	2.3.2.1a	98.52%
M	GISAID EpiFlu	EPI2062119 A/duck/Bangladesh/19D1817/2021(H5N1)	2.3.4.4b	99%
	NCBI nt	LC765296 A/crow/Fukuoka/TU48–37/2022(H5N1))	2.3.4.4b	99.59%
NS	GISAID EpiFlu	EPI3360023 A/duck/Bangladesh/18-D-2109/2023(H?N?)	2.3.2.1a	99%
	NCBI nt	OP023561 A/chicken/Bangladesh/50585/2021(H5N1)	2.3.2.1a	98.93%

*HA, hemagglutinin; M, matrix; NA, neuraminidase; NP, nucleoprotein; NS, nonstructural; PA, polymerase acidic; PB1, polymerase basic 1; PB2, polymerase basic 2.

Appendix Figure 1 (following pages). Detailed phylogenetic analysis of genes of A/Victoria/149/2024. Maximum-likelihood nucleotide trees are shown for A) PB2, B) PB1, C) PA, D) HA, E) NP, F) NA, G) MP, H) NS genes with a sample of BLAST-matched nucleotide sequences available (up to 21 May 2024) from GISAID and GenBank. Bootstrap values >90% for key nodes are shown. Scale bars indicate the number of nucleotide substitutions per site for each gene. HA, hemagglutinin; MP, matrix protein; NA, neuraminidase; NP, nucleoprotein; NS, nonstructural; PA, polymerase acidic; PB1, polymerase basic 1; PB2, polymerase basic 2.

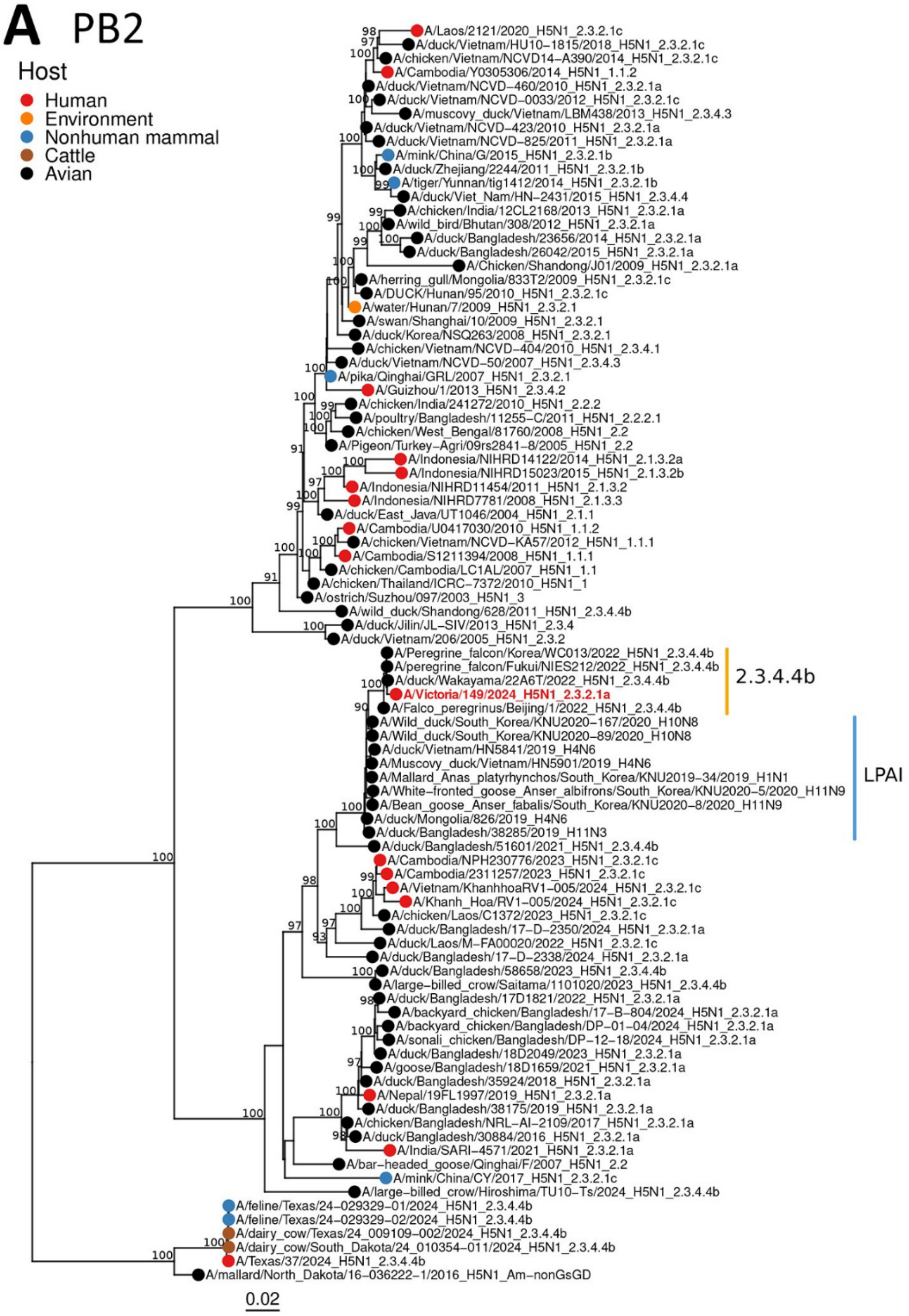
A PB2
Host
Human
Environment
Nonhuman mammal
Cattle
Avian
A/Laos/2121/2020_H5N1_2.3.2.1c
A/duck/Vietnam/HU10-1815/2018_H5N1_2.3.2.1c
A/chicken/Vietnam/NCVD14-A390/2014_H5N1_2.3.2.1c
A/Cambodia/Y0305306/2014_H5N1_1.1.2
A/duck/Vietnam/NCVD-460/2010_H5N1_2.3.2.1a
A/duck/Vietnam/NCVD-0033/2012_H5N1_2.3.2.1c
A/muscovy_duck/Vietnam/LBM438/2013_H5N1_2.3.4.3
A/duck/Vietnam/NCVD-423/2010_H5N1_2.3.2.1a
A/duck/Vietnam/NCVD-825/2011_H5N1_2.3.2.1a
A/mink/China/G/2015_H5N1_2.3.2.1b
A/duck/Zhejiang/2244/2011_H5N1_2.3.2.1b
A/tiger/Yunnan/tig1412/2014_H5N1_2.3.2.1b
A/duck/Viet_Nam/HN-2431/2015_H5N1_2.3.4.4
A/chicken/India/12CL2168/2013_H5N1_2.3.2.1a
A/wild_bird/Bhutan/308/2012_H5N1_2.3.2.1a
A/duck/Bangladesh/23656/2014_H5N1_2.3.2.1a
A/duck/Bangladesh/26042/2015_H5N1_2.3.2.1a
A/Chicken/Shandong/J01/2009_H5N1_2.3.2.1a
A/herring_gull/Mongolia/833T2/2009_H5N1_2.3.2.1c
A/DUCK/Hunan/95/2010_H5N1_2.3.2.1c
A/water/Hunan/7/2009_H5N1_2.3.2.1
A/swan/Shanghai/10/2009_H5N1_2.3.2.1
A/duck/Korea/NSQ263/2008_H5N1_2.3.2.1
A/chicken/Vietnam/NCVD-404/2010_H5N1_2.3.4.1
A/duck/Vietnam/NCVD-50/2007_H5N1_2.3.4.3
A/pika/Qinghai/GRL/2007_H5N1_2.3.2.1
A/Guizhou/1/2013_H5N1_2.3.4.2
A/chicken/India/241272/2010_H5N1_2.2.2
A/poultry/Bangladesh/11255-C/2011_H5N1_2.2.2.1
A/chicken/West_Bengal/81760/2008_H5N1_2.2
A/Pigeon/Turkey-Agri/09rs2841-8/2005_H5N1_2.2
A/Indonesia/NIHRD14122/2014_H5N1_2.1.3.2a
A/Indonesia/NIHRD15023/2015_H5N1_2.1.3.2b
A/Indonesia/NIHRD11454/2011_H5N1_2.1.3.2
A/Indonesia/NIHRD7781/2008_H5N1_2.1.3.3
A/duck/East_Java/UT1046/2004_H5N1_2.1.1
A/Cambodia/U0417030/2010_H5N1_1.1.2
A/chicken/Vietnam/NCVD-KA57/2012_H5N1_1.1.1
A/Cambodia/S1211394/2008_H5N1_1.1.1
A/chicken/Cambodia/LC1AL/2007_H5N1_1.1
A/chicken/Thailand/ICRC-7372/2010_H5N1_1
A/ostrich/Suzhou/097/2003_H5N1_3
A/wild_duck/Shandong/628/2011_H5N1_2.3.4.4b
A/duck/Jilin/JL-SIV/2013_H5N1_2.3.4
A/duck/Vietnam/206/2005_H5N1_2.3.2
A/Peregrine_falcon/Korea/WC013/2022_H5N1_2.3.4.4b
A/peregrine_falcon/Fukui/NIES212/2022_H5N1_2.3.4.4b
A/duck/Wakayama/22A6T/2022_H5N1_2.3.4.4b
A/Victoria/149/2024_H5N1_2.3.2.1a
A/Falco_peregrinus/Beijing/1/2022_H5N1_2.3.4.4b
2.3.4.4b
A/Wild_duck/South_Korea/KNU2020-167/2020_H10N8
A/Wild_duck/South_Korea/KNU2020-89/2020_H10N8
A/duck/Vietnam/HN5841/2019_H4N6
A/Muscovy_duck/Vietnam/HN5901/2019_H4N6
A/Mallard_Anas_platyrhynchos/South_Korea/KNU2019-34/2019_H1N1
A/White-fronted_goose_Anser_albifrons/South_Korea/KNU2020-5/2020_H11N9
A/Bean_goose_Anser_fabalis/South_Korea/KNU2020-8/2020_H11N9
A/duck/Mongolia/826/2019_H4N6
A/duck/Bangladesh/38285/2019_H11N3
LPAI
A/duck/Bangladesh/51601/2021_H5N1_2.3.4.4b
A/Cambodia/NPH230776/2023_H5N1_2.3.2.1c
A/Cambodia/2311257/2023_H5N1_2.3.2.1c
A/Vietnam/KhanhhoaRV1-005/2024_H5N1_2.3.2.1c
A/Khanh_Hoa/RV1-005/2024_H5N1_2.3.2.1c
A/chicken/Laos/C1372/2023_H5N1_2.3.2.1c
A/duck/Bangladesh/17-D-2350/2024_H5N1_2.3.2.1a
A/duck/Laos/M-FA00020/2022_H5N1_2.3.2.1c
A/duck/Bangladesh/17-D-2338/2024_H5N1_2.3.2.1a
A/duck/Bangladesh/58658/2023_H5N1_2.3.4.4b
A/large-billed_crow/Saitama/1101020/2023_H5N1_2.3.4.4b
A/duck/Bangladesh/17D1821/2022_H5N1_2.3.2.1a
A/backyard_chicken/Bangladesh/17-B-804/2024_H5N1_2.3.2.1a
A/backyard_chicken/Bangladesh/DP-01-04/2024_H5N1_2.3.2.1a
A/sonali_chicken/Bangladesh/DP-12-18/2024_H5N1_2.3.2.1a
A/duck/Bangladesh/18D2049/2023_H5N1_2.3.2.1a
A/goose/Bangladesh/18D1659/2021_H5N1_2.3.2.1a
A/duck/Bangladesh/35924/2018_H5N1_2.3.2.1a
A/Nepal/19FL1997/2019_H5N1_2.3.2.1a
A/duck/Bangladesh/38175/2019_H5N1_2.3.2.1a
A/chicken/Bangladesh/NRL-AI-2109/2017_H5N1_2.3.2.1a
A/duck/Bangladesh/30884/2016_H5N1_2.3.2.1a
A/India/SARI-4571/2021_H5N1_2.3.2.1a
A/bar-headed_goose/Qinghai/F/2007_H5N1_2.2
A/mink/China/CY/2017_H5N1_2.3.2.1c
A/large-billed_crow/Hiroshima/TU10-Ts/2024_H5N1_2.3.4.4b
A/feline/Texas/24-029329-01/2024_H5N1_2.3.4.4b
A/feline/Texas/24-029329-02/2024_H5N1_2.3.4.4b
A/dairy_cow/Texas/24_009109-002/2024_H5N1_2.3.4.4b
A/dairy_cow/South_Dakota/24_010354-011/2024_H5N1_2.3.4.4b
A/Texas/37/2024_H5N1_2.3.4.4b
A/mallard/North_Dakota/16-036222-1/2016_H5N1_Am-nonGsGD
0.02

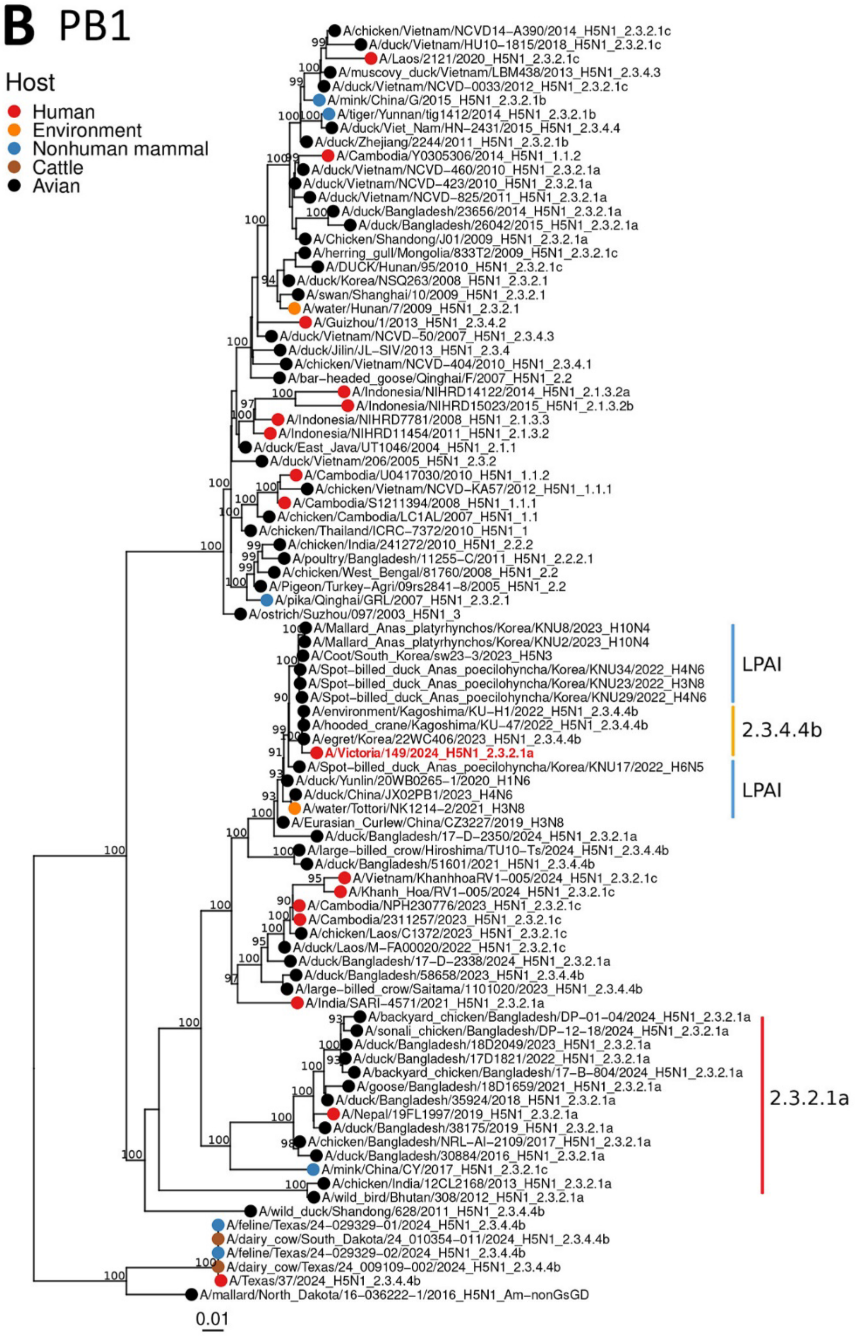

B PB1
Host
Human
Environment
Nonhuman mammal
Cattle
Avian
A/chicken/Vietnam/NCVD14–A390/2014_H5N1_2.3.2.1c
A/duck/Vietnam/HU10–1815/2018_H5N1_2.3.2.1c
A/Laos/2121/2020_H5N1_2.3.2.1c
A/muscovy_duck/Vietnam/LBM438/2013_H5N1_2.3.4.3
A/duck/Vietnam/NCVD–0033/2012_H5N1_2.3.2.1c
A/mink/China/G/2015_H5N1_2.3.2.1b
A/tiger/Yunnan/tig1412/2014_H5N1_2.3.2.1b
A/duck/Viet_Nam/HN–2431/2015_H5N1_2.3.4.4
A/duck/Zhejiang/2244/2011_H5N1_2.3.2.1b
A/Cambodia/Y0305306/2014_H5N1_1.1.2
A/duck/Vietnam/NCVD–460/2010_H5N1_2.3.2.1a
A/duck/Vietnam/NCVD–423/2010_H5N1_2.3.2.1a
A/duck/Vietnam/NCVD–825/2011_H5N1_2.3.2.1a
A/duck/Bangladesh/23656/2014_H5N1_2.3.2.1a
A/duck/Bangladesh/26042/2015_H5N1_2.3.2.1a
A/Chicken/Shandong/J01/2009_H5N1_2.3.2.1a
A/herring_gull/Mongolia/833T2/2009_H5N1_2.3.2.1c
A/DUCK/Hunan/95/2010_H5N1_2.3.2.1c
A/duck/Korea/NSQ263/2008_H5N1_2.3.2.1
A/swan/Shanghai/10/2009_H5N1_2.3.2.1
A/water/Hunan/7/2009_H5N1_2.3.2.1
A/Guizhou/1/2013_H5N1_2.3.4.2
A/duck/Vietnam/NCVD–50/2007_H5N1_2.3.4.3
A/duck/Jilin/JL–SIV/2013_H5N1_2.3.4
A/chicken/Vietnam/NCVD–404/2010_H5N1_2.3.4.1
A/bar–headed_goose/Qinghai/F/2007_H5N1_2.2
A/Indonesia/NIHRD14122/2014_H5N1_2.1.3.2a
A/Indonesia/NIHRD15023/2015_H5N1_2.1.3.2b
A/Indonesia/NIHRD7781/2008_H5N1_2.1.3.3
A/Indonesia/NIHRD11454/2011_H5N1_2.1.3.2
A/duck/East_Java/UT1046/2004_H5N1_2.1.1
A/duck/Vietnam/206/2005_H5N1_2.3.2
A/Cambodia/U0417030/2010_H5N1_1.1.2
A/chicken/Vietnam/NCVD–KA57/2012_H5N1_1.1.1
A/Cambodia/S1211394/2008_H5N1_1.1.1
A/chicken/Cambodia/LC1AL/2007_H5N1_1.1
A/chicken/Thailand/ICRC–7372/2010_H5N1_1
A/chicken/India/241272/2010_H5N1_2.2.2
A/poultry/Bangladesh/11255–C/2011_H5N1_2.2.2.1
A/chicken/West_Bengal/81760/2008_H5N1_2.2
A/Pigeon/Turkey–Agri/09rs2841–8/2005_H5N1_2.2
A/pika/Qinghai/GRL/2007_H5N1_2.3.2.1
A/ostrich/Suzhou/097/2003_H5N1_3
A/Mallard_Anas_platyrhynchos/Korea/KNU8/2023_H10N4
A/Mallard_Anas_platyrhynchos/Korea/KNU2/2023_H10N4
A/Coot/South_Korea/sw23–3/2023_H5N3
A/Spot–billed_duck_Anas_poecilohyncha/Korea/KNU34/2022_H4N6
A/Spot–billed_duck_Anas_poecilohyncha/Korea/KNU23/2022_H3N8
A/Spot–billed_duck_Anas_poecilohyncha/Korea/KNU29/2022_H4N6
A/environment/Kagoshima/KU–H1/2022_H5N1_2.3.4.4b
A/hooded_crane/Kagoshima/KU–47/2022_H5N1_2.3.4.4b
A/egret/Korea/22WC406/2023_H5N1_2.3.4.4b
A/Victoria/149/2024_H5N1_2.3.2.1a
A/Spot–billed_duck_Anas_poecilohyncha/Korea/KNU17/2022_H6N5
A/duck/Yunlin/20WB0265–1/2020_H1N6
A/duck/China/JX02PB1/2023_H4N6
A/water/Tottori/NK1214–2/2021_H3N8
A/Eurasian_Curlew/China/CZ3227/2019_H3N8
A/duck/Bangladesh/17–D–2350/2024_H5N1_2.3.2.1a
A/large–billed_crow/Hiroshima/TU10–Ts/2024_H5N1_2.3.4.4b
A/duck/Bangladesh/51601/2021_H5N1_2.3.4.4b
A/Vietnam/KhanhhoaRV1–005/2024_H5N1_2.3.2.1c
A/Khanh_Hoa/RV1–005/2024_H5N1_2.3.2.1c
A/Cambodia/NPH230776/2023_H5N1_2.3.2.1c
A/Cambodia/2311257/2023_H5N1_2.3.2.1c
A/chicken/Laos/C1372/2023_H5N1_2.3.2.1c
A/duck/Laos/M–FA00020/2022_H5N1_2.3.2.1c
A/duck/Bangladesh/17–D–2338/2024_H5N1_2.3.2.1a
A/duck/Bangladesh/58658/2023_H5N1_2.3.4.4b
A/large–billed_crow/Saitama/1101020/2023_H5N1_2.3.4.4b
A/India/SARI–4571/2021_H5N1_2.3.2.1a
A/backyard_chicken/Bangladesh/DP–01–04/2024_H5N1_2.3.2.1a
A/sonali_chicken/Bangladesh/DP–12–18/2024_H5N1_2.3.2.1a
A/duck/Bangladesh/18D2049/2023_H5N1_2.3.2.1a
A/duck/Bangladesh/17D1821/2022_H5N1_2.3.2.1a
A/backyard_chicken/Bangladesh/17–B–804/2024_H5N1_2.3.2.1a
A/goose/Bangladesh/18D1659/2021_H5N1_2.3.2.1a
A/duck/Bangladesh/35924/2018_H5N1_2.3.2.1a
A/Nepal/19FL1997/2019_H5N1_2.3.2.1a
A/duck/Bangladesh/38175/2019_H5N1_2.3.2.1a
A/chicken/Bangladesh/NRL–AI–2109/2017_H5N1_2.3.2.1a
A/duck/Bangladesh/30884/2016_H5N1_2.3.2.1a
A/mink/China/CY/2017_H5N1_2.3.2.1c
A/chicken/India/12CL2168/2013_H5N1_2.3.2.1a
A/wild_bird/Bhutan/308/2012_H5N1_2.3.2.1a
A/wild_duck/Shandong/628/2011_H5N1_2.3.4.4b
A/feline/Texas/24–029329–01/2024_H5N1_2.3.4.4b
A/dairy_cow/South_Dakota/24_010354–011/2024_H5N1_2.3.4.4b
A/feline/Texas/24–029329–02/2024_H5N1_2.3.4.4b
A/dairy_cow/Texas/24_009109–002/2024_H5N1_2.3.4.4b
A/Texas/37/2024_H5N1_2.3.4.4b
A/mallard/North_Dakota/16–036222–1/2016_H5N1_Am–nonGsGD
LPAI
2.3.4.4b
LPAI
2.3.2.1a
0.01

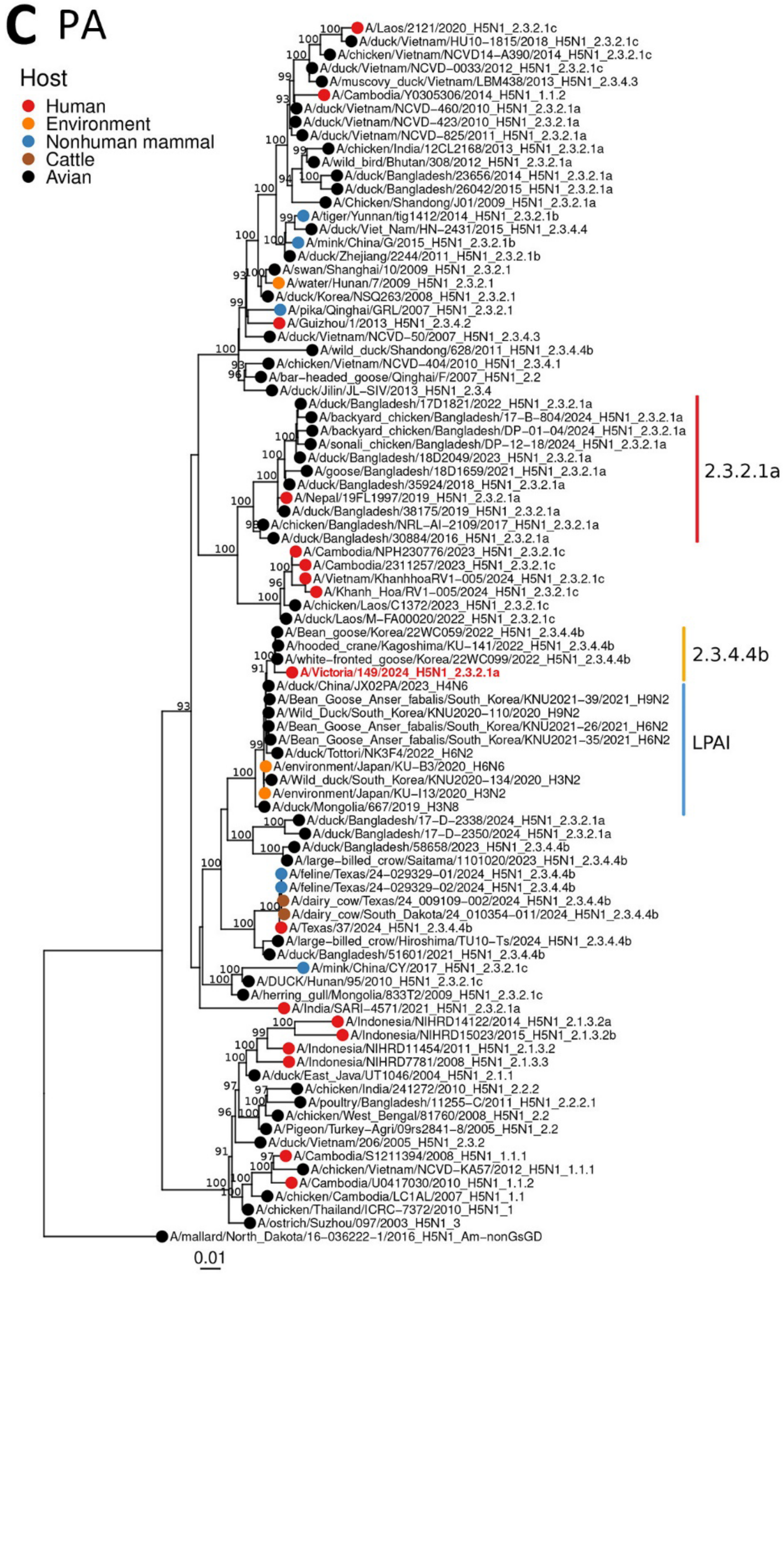
C PA
Host
Human
Environment
Nonhuman mammal
Cattle
Avian
A/Laos/2121/2020_H5N1_2.3.2.1c
A/duck/Vietnam/HU10-1815/2018_H5N1_2.3.2.1c
A/chicken/Vietnam/NCVD14-A390/2014_H5N1_2.3.2.1c
A/duck/Vietnam/NCVD-0033/2012_H5N1_2.3.2.1c
A/muscovy_duck/Vietnam/LBM438/2013_H5N1_2.3.4.3
A/Cambodia/Y0305306/2014_H5N1_1.1.2
A/duck/Vietnam/NCVD-460/2010_H5N1_2.3.2.1a
A/duck/Vietnam/NCVD-423/2010_H5N1_2.3.2.1a
A/duck/Vietnam/NCVD-825/2011_H5N1_2.3.2.1a
A/chicken/India/12CL2168/2013_H5N1_2.3.2.1a
A/wild_bird/Bhutan/308/2012_H5N1_2.3.2.1a
A/duck/Bangladesh/23656/2014_H5N1_2.3.2.1a
A/duck/Bangladesh/26042/2015_H5N1_2.3.2.1a
A/Chicken/Shandong/J01/2009_H5N1_2.3.2.1a
A/tiger/Yunnan/tig1412/2014_H5N1_2.3.2.1b
A/duck/Viet_Nam/HN-2431/2015_H5N1_2.3.4.4
A/mink/China/G/2015_H5N1_2.3.2.1b
A/duck/Zhejiang/2244/2011_H5N1_2.3.2.1b
A/swan/Shanghai/10/2009_H5N1_2.3.2.1
A/water/Hunan/7/2009_H5N1_2.3.2.1
A/duck/Korea/NSQ263/2008_H5N1_2.3.2.1
A/pika/Qinghai/GRL/2007_H5N1_2.3.2.1
A/Guizhou/1/2013_H5N1_2.3.4.2
A/duck/Vietnam/NCVD-50/2007_H5N1_2.3.4.3
A/wild_duck/Shandong/628/2011_H5N1_2.3.4.4b
A/chicken/Vietnam/NCVD-404/2010_H5N1_2.3.4.1
A/bar-headed_goose/Qinghai/F/2007_H5N1_2.2
A/duck/Jilin/JL-SIV/2013_H5N1_2.3.4
A/duck/Bangladesh/17D1821/2022_H5N1_2.3.2.1a
A/backyard_chicken/Bangladesh/17-B-804/2024_H5N1_2.3.2.1a
A/backyard_chicken/Bangladesh/DP-01-04/2024_H5N1_2.3.2.1a
A/sonali_chicken/Bangladesh/DP-12-18/2024_H5N1_2.3.2.1a
A/duck/Bangladesh/18D2049/2023_H5N1_2.3.2.1a
A/goose/Bangladesh/18D1659/2021_H5N1_2.3.2.1a
A/duck/Bangladesh/35924/2018_H5N1_2.3.2.1a
A/Nepal/19FL1997/2019_H5N1_2.3.2.1a
A/duck/Bangladesh/38175/2019_H5N1_2.3.2.1a
A/chicken/Bangladesh/NRL-AI-2109/2017_H5N1_2.3.2.1a
A/duck/Bangladesh/30884/2016_H5N1_2.3.2.1a
2.3.2.1a
A/Cambodia/NPH230776/2023_H5N1_2.3.2.1c
A/Cambodia/2311257/2023_H5N1_2.3.2.1c
A/Vietnam/KhanhhoaRV1-005/2024_H5N1_2.3.2.1c
A/Khanh_Hoa/RV1-005/2024_H5N1_2.3.2.1c
A/chicken/Laos/C1372/2023_H5N1_2.3.2.1c
A/duck/Laos/M-FA00020/2022_H5N1_2.3.2.1c
A/Bean_goose/Korea/22WC059/2022_H5N1_2.3.4.4b
A/hooded_crane/Kagoshima/KU-141/2022_H5N1_2.3.4.4b
A/white-fronted_goose/Korea/22WC099/2022_H5N1_2.3.4.4b
A/Victoria/149/2024_H5N1_2.3.2.1a
2.3.4.4b
A/duck/China/JX02PA/2023_H4N6
A/Bean_Goose_Anser_fabalis/South_Korea/KNU2021-39/2021_H9N2
A/Wild_Duck/South_Korea/KNU2020-110/2020_H9N2
A/Bean_Goose_Anser_fabalis/South_Korea/KNU2021-26/2021_H6N2
A/Bean_Goose_Anser_fabalis/South_Korea/KNU2021-35/2021_H6N2
A/duck/Tottori/NK3F4/2022_H6N2
A/environment/Japan/KU-B3/2020_H6N6
A/Wild_duck/South_Korea/KNU2020-134/2020_H3N2
A/environment/Japan/KU-I13/2020_H3N2
A/duck/Mongolia/667/2019_H3N8
LPAI
A/duck/Bangladesh/17-D-2338/2024_H5N1_2.3.2.1a
A/duck/Bangladesh/17-D-2350/2024_H5N1_2.3.2.1a
A/duck/Bangladesh/58658/2023_H5N1_2.3.4.4b
A/large-billed_crow/Saitama/1101020/2023_H5N1_2.3.4.4b
A/feline/Texas/24-029329-01/2024_H5N1_2.3.4.4b
A/feline/Texas/24-029329-02/2024_H5N1_2.3.4.4b
A/dairy_cow/Texas/24_009109-002/2024_H5N1_2.3.4.4b
A/dairy_cow/South_Dakota/24_010354-011/2024_H5N1_2.3.4.4b
A/Texas/37/2024_H5N1_2.3.4.4b
A/large-billed_crow/Hiroshima/TU10-Ts/2024_H5N1_2.3.4.4b
A/duck/Bangladesh/51601/2021_H5N1_2.3.4.4b
A/mink/China/CY/2017_H5N1_2.3.2.1c
A/DUCK/Hunan/95/2010_H5N1_2.3.2.1c
A/herring_gull/Mongolia/833T2/2009_H5N1_2.3.2.1c
A/India/SARI-4571/2021_H5N1_2.3.2.1a
A/Indonesia/NIHRD14122/2014_H5N1_2.1.3.2a
A/Indonesia/NIHRD15023/2015_H5N1_2.1.3.2b
A/Indonesia/NIHRD11454/2011_H5N1_2.1.3.2
A/Indonesia/NIHRD7781/2008_H5N1_2.1.3.3
A/duck/East_Java/UT1046/2004_H5N1_2.1.1
A/chicken/India/241272/2010_H5N1_2.2.2
A/poultry/Bangladesh/11255-C/2011_H5N1_2.2.2.1
A/chicken/West_Bengal/81760/2008_H5N1_2.2
A/Pigeon/Turkey-Agri/09rs2841-8/2005_H5N1_2.2
A/duck/Vietnam/206/2005_H5N1_2.3.2
A/Cambodia/S1211394/2008_H5N1_1.1.1
A/chicken/Vietnam/NCVD-KA57/2012_H5N1_1.1.1
A/Cambodia/U0417030/2010_H5N1_1.1.2
A/chicken/Cambodia/LC1AL/2007_H5N1_1.1
A/chicken/Thailand/ICRC-7372/2010_H5N1_1
A/ostrich/Suzhou/097/2003_H5N1_3
A/mallard/North_Dakota/16-036222-1/2016_H5N1_Am-nonGsGD
0.01

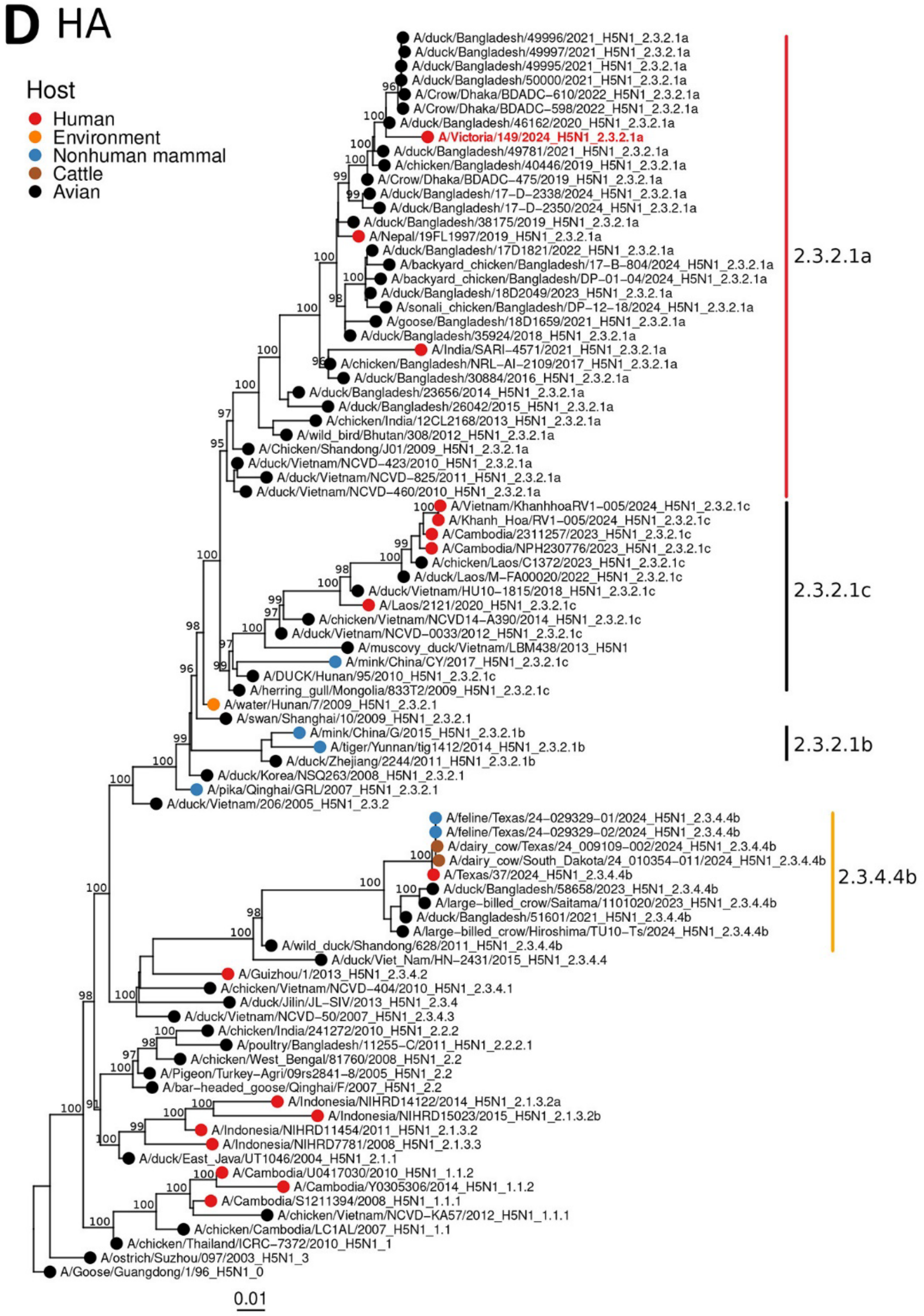
D HA
Host
Human
Environment
Nonhuman mammal
Cattle
Avian
A/duck/Bangladesh/49996/2021_H5N1_2.3.2.1a
A/duck/Bangladesh/49997/2021_H5N1_2.3.2.1a
A/duck/Bangladesh/49995/2021_H5N1_2.3.2.1a
A/duck/Bangladesh/50000/2021_H5N1_2.3.2.1a
A/Crow/Dhaka/BDADC-610/2022_H5N1_2.3.2.1a
A/Crow/Dhaka/BDADC-598/2022_H5N1_2.3.2.1a
A/duck/Bangladesh/46162/2020_H5N1_2.3.2.1a
A/Victoria/149/2024_H5N1_2.3.2.1a
A/duck/Bangladesh/49781/2021_H5N1_2.3.2.1a
A/chicken/Bangladesh/40446/2019_H5N1_2.3.2.1a
A/Crow/Dhaka/BDADC-475/2019_H5N1_2.3.2.1a
A/duck/Bangladesh/17-D-2338/2024_H5N1_2.3.2.1a
A/duck/Bangladesh/17-D-2350/2024_H5N1_2.3.2.1a
A/duck/Bangladesh/38175/2019_H5N1_2.3.2.1a
A/Nepal/19FL1997/2019_H5N1_2.3.2.1a
A/duck/Bangladesh/17D1821/2022_H5N1_2.3.2.1a
A/backyard_chicken/Bangladesh/17-B-804/2024_H5N1_2.3.2.1a
A/backyard_chicken/Bangladesh/DP-01-04/2024_H5N1_2.3.2.1a
A/duck/Bangladesh/18D2049/2023_H5N1_2.3.2.1a
A/sonali_chicken/Bangladesh/DP-12-18/2024_H5N1_2.3.2.1a
A/goose/Bangladesh/18D1659/2021_H5N1_2.3.2.1a
A/duck/Bangladesh/35924/2018_H5N1_2.3.2.1a
A/India/SARI-4571/2021_H5N1_2.3.2.1a
A/chicken/Bangladesh/NRL-AI-2109/2017_H5N1_2.3.2.1a
A/duck/Bangladesh/30884/2016_H5N1_2.3.2.1a
A/duck/Bangladesh/23656/2014_H5N1_2.3.2.1a
A/duck/Bangladesh/26042/2015_H5N1_2.3.2.1a
A/chicken/India/12CL2168/2013_H5N1_2.3.2.1a
A/wild_bird/Bhutan/308/2012_H5N1_2.3.2.1a
A/Chicken/Shandong/J01/2009_H5N1_2.3.2.1a
A/duck/Vietnam/NCVD-423/2010_H5N1_2.3.2.1a
A/duck/Vietnam/NCVD-825/2011_H5N1_2.3.2.1a
A/duck/Vietnam/NCVD-460/2010_H5N1_2.3.2.1a
2.3.2.1a
A/Vietnam/KhanhhoaRV1-005/2024_H5N1_2.3.2.1c
A/Khanh_Hoa/RV1-005/2024_H5N1_2.3.2.1c
A/Cambodia/2311257/2023_H5N1_2.3.2.1c
A/Cambodia/NPH230776/2023_H5N1_2.3.2.1c
A/chicken/Laos/C1372/2023_H5N1_2.3.2.1c
A/duck/Laos/M-FA00020/2022_H5N1_2.3.2.1c
A/duck/Vietnam/HU10-1815/2018_H5N1_2.3.2.1c
A/Laos/2121/2020_H5N1_2.3.2.1c
A/chicken/Vietnam/NCVD14-A390/2014_H5N1_2.3.2.1c
A/duck/Vietnam/NCVD-0033/2012_H5N1_2.3.2.1c
A/muscovy_duck/Vietnam/LBM438/2013_H5N1
A/mink/China/CY/2017_H5N1_2.3.2.1c
A/DUCK/Hunan/95/2010_H5N1_2.3.2.1c
A/herring_gull/Mongolia/833T2/2009_H5N1_2.3.2.1c
2.3.2.1c
A/water/Hunan/7/2009_H5N1_2.3.2.1
A/swan/Shanghai/10/2009_H5N1_2.3.2.1
A/mink/China/G/2015_H5N1_2.3.2.1b
A/tiger/Yunnan/tig1412/2014_H5N1_2.3.2.1b
A/duck/Zhejiang/2244/2011_H5N1_2.3.2.1b
2.3.2.1b
A/duck/Korea/NSQ263/2008_H5N1_2.3.2.1
A/pika/Qinghai/GRL/2007_H5N1_2.3.2.1
A/duck/Vietnam/206/2005_H5N1_2.3.2
A/feline/Texas/24-029329-01/2024_H5N1_2.3.4.4b
A/feline/Texas/24-029329-02/2024_H5N1_2.3.4.4b
A/dairy_cow/Texas/24_009109-002/2024_H5N1_2.3.4.4b
A/dairy_cow/South_Dakota/24_010354-011/2024_H5N1_2.3.4.4b
A/Texas/37/2024_H5N1_2.3.4.4b
A/duck/Bangladesh/58658/2023_H5N1_2.3.4.4b
A/large-billed_crow/Saitama/1101020/2023_H5N1_2.3.4.4b
A/duck/Bangladesh/51601/2021_H5N1_2.3.4.4b
A/large-billed_crow/Hiroshima/TU10-Ts/2024_H5N1_2.3.4.4b
A/wild_duck/Shandong/628/2011_H5N1_2.3.4.4b
A/duck/Viet_Nam/HN-2431/2015_H5N1_2.3.4.4
2.3.4.4b
A/Guizhou/1/2013_H5N1_2.3.4.2
A/chicken/Vietnam/NCVD-404/2010_H5N1_2.3.4.1
A/duck/Jilin/JL-SIV/2013_H5N1_2.3.4
A/duck/Vietnam/NCVD-50/2007_H5N1_2.3.4.3
A/chicken/India/241272/2010_H5N1_2.2.2
A/poultry/Bangladesh/11255-C/2011_H5N1_2.2.2.1
A/chicken/West_Bengal/81760/2008_H5N1_2.2
A/Pigeon/Turkey-Agri/09rs2841-8/2005_H5N1_2.2
A/bar-headed_goose/Qinghai/F/2007_H5N1_2.2
A/Indonesia/NIHRD14122/2014_H5N1_2.1.3.2a
A/Indonesia/NIHRD15023/2015_H5N1_2.1.3.2b
A/Indonesia/NIHRD11454/2011_H5N1_2.1.3.2
A/Indonesia/NIHRD7781/2008_H5N1_2.1.3.3
A/duck/East_Java/UT1046/2004_H5N1_2.1.1
A/Cambodia/U0417030/2010_H5N1_1.1.2
A/Cambodia/Y0305306/2014_H5N1_1.1.2
A/Cambodia/S1211394/2008_H5N1_1.1.1
A/chicken/Vietnam/NCVD-KA57/2012_H5N1_1.1.1
A/chicken/Cambodia/LC1AL/2007_H5N1_1.1
A/chicken/Thailand/ICRC-7372/2010_H5N1_1
A/ostrich/Suzhou/097/2003_H5N1_3
A/Goose/Guangdong/1/96_H5N1_0
0.01

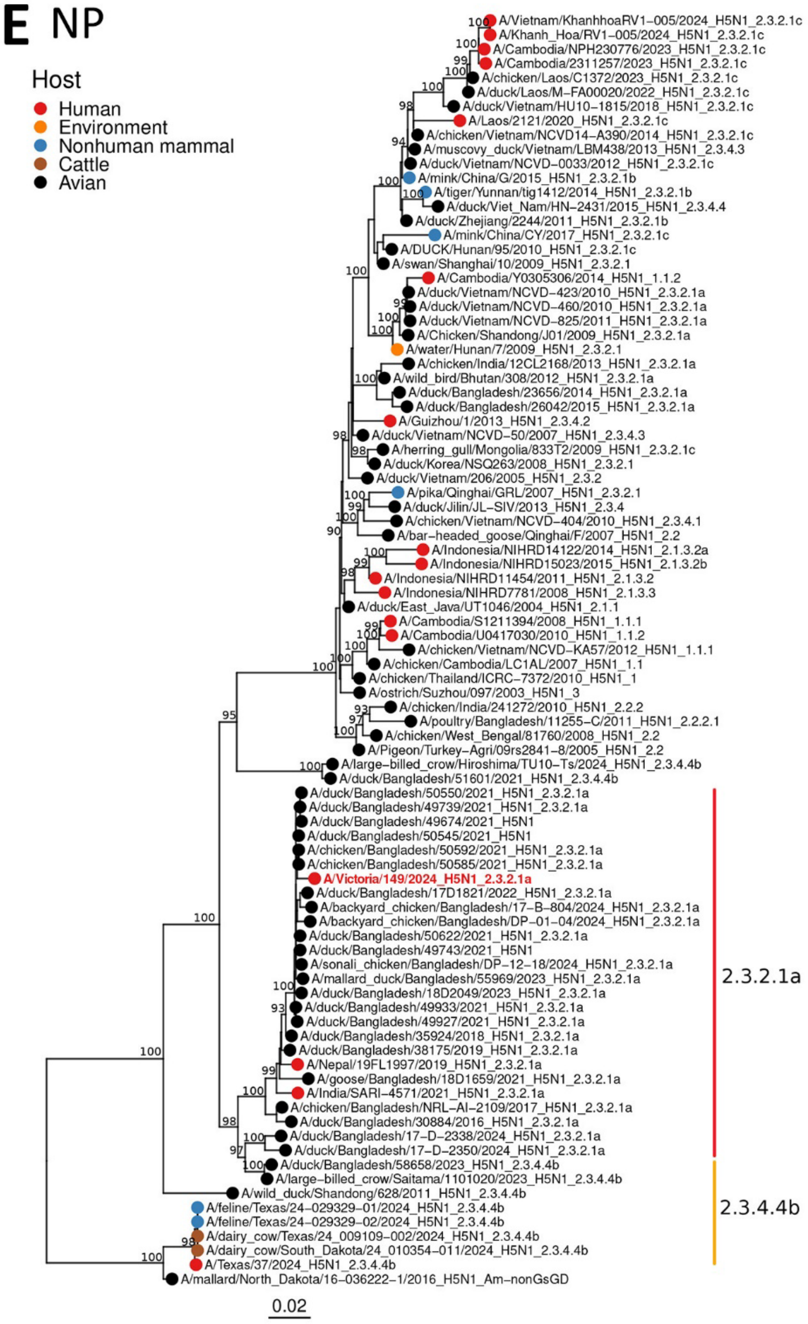
E NP
Host
Human
Environment
Nonhuman mammal
Cattle
Avian
A/Vietnam/KhanhhoaRV1−005/2024_H5N1_2.3.2.1c
A/Khanh_Hoa/RV1−005/2024_H5N1_2.3.2.1c
A/Cambodia/NPH230776/2023_H5N1_2.3.2.1c
A/Cambodia/2311257/2023_H5N1_2.3.2.1c
A/chicken/Laos/C1372/2023_H5N1_2.3.2.1c
A/duck/Laos/M−FA00020/2022_H5N1_2.3.2.1c
A/duck/Vietnam/HU10−1815/2018_H5N1_2.3.2.1c
A/Laos/2121/2020_H5N1_2.3.2.1c
A/chicken/Vietnam/NCVD14−A390/2014_H5N1_2.3.2.1c
A/muscovy_duck/Vietnam/LBM438/2013_H5N1_2.3.4.3
A/duck/Vietnam/NCVD−0033/2012_H5N1_2.3.2.1c
A/mink/China/G/2015_H5N1_2.3.2.1b
A/tiger/Yunnan/tig1412/2014_H5N1_2.3.2.1b
A/duck/Viet_Nam/HN−2431/2015_H5N1_2.3.4.4
A/duck/Zhejiang/2244/2011_H5N1_2.3.2.1b
A/mink/China/CY/2017_H5N1_2.3.2.1c
A/DUCK/Hunan/95/2010_H5N1_2.3.2.1c
A/swan/Shanghai/10/2009_H5N1_2.3.2.1
A/Cambodia/Y0305306/2014_H5N1_1.1.2
A/duck/Vietnam/NCVD−423/2010_H5N1_2.3.2.1a
A/duck/Vietnam/NCVD−460/2010_H5N1_2.3.2.1a
A/duck/Vietnam/NCVD−825/2011_H5N1_2.3.2.1a
A/Chicken/Shandong/J01/2009_H5N1_2.3.2.1a
A/water/Hunan/7/2009_H5N1_2.3.2.1
A/chicken/India/12CL2168/2013_H5N1_2.3.2.1a
A/wild_bird/Bhutan/308/2012_H5N1_2.3.2.1a
A/duck/Bangladesh/23656/2014_H5N1_2.3.2.1a
A/duck/Bangladesh/26042/2015_H5N1_2.3.2.1a
A/Guizhou/1/2013_H5N1_2.3.4.2
A/duck/Vietnam/NCVD−50/2007_H5N1_2.3.4.3
A/herring_gull/Mongolia/833T2/2009_H5N1_2.3.2.1c
A/duck/Korea/NSQ263/2008_H5N1_2.3.2.1
A/duck/Vietnam/206/2005_H5N1_2.3.2
A/pika/Qinghai/GRL/2007_H5N1_2.3.2.1
A/duck/Jilin/JL−SIV/2013_H5N1_2.3.4
A/chicken/Vietnam/NCVD−404/2010_H5N1_2.3.4.1
A/bar−headed_goose/Qinghai/F/2007_H5N1_2.2
A/Indonesia/NIHRD14122/2014_H5N1_2.1.3.2a
A/Indonesia/NIHRD15023/2015_H5N1_2.1.3.2b
A/Indonesia/NIHRD11454/2011_H5N1_2.1.3.2
A/Indonesia/NIHRD7781/2008_H5N1_2.1.3.3
A/duck/East_Java/UT1046/2004_H5N1_2.1.1
A/Cambodia/S1211394/2008_H5N1_1.1.1
A/Cambodia/U0417030/2010_H5N1_1.1.2
A/chicken/Vietnam/NCVD−KA57/2012_H5N1_1.1.1
A/chicken/Cambodia/LC1AL/2007_H5N1_1.1
A/chicken/Thailand/ICRC−7372/2010_H5N1_1
A/ostrich/Suzhou/097/2003_H5N1_3
A/chicken/India/241272/2010_H5N1_2.2.2
A/poultry/Bangladesh/11255−C/2011_H5N1_2.2.2.1
A/chicken/West_Bengal/81760/2008_H5N1_2.2
A/Pigeon/Turkey−Agri/09rs2841−8/2005_H5N1_2.2
A/large−billed_crow/Hiroshima/TU10−Ts/2024_H5N1_2.3.4.4b
A/duck/Bangladesh/51601/2021_H5N1_2.3.4.4b
A/duck/Bangladesh/50550/2021_H5N1_2.3.2.1a
A/duck/Bangladesh/49739/2021_H5N1_2.3.2.1a
A/duck/Bangladesh/49674/2021_H5N1
A/duck/Bangladesh/50545/2021_H5N1
A/chicken/Bangladesh/50592/2021_H5N1_2.3.2.1a
A/chicken/Bangladesh/50585/2021_H5N1_2.3.2.1a
A/Victoria/149/2024_H5N1_2.3.2.1a
A/duck/Bangladesh/17D1821/2022_H5N1_2.3.2.1a
A/backyard_chicken/Bangladesh/17−B−804/2024_H5N1_2.3.2.1a
A/backyard_chicken/Bangladesh/DP−01−04/2024_H5N1_2.3.2.1a
A/duck/Bangladesh/50622/2021_H5N1_2.3.2.1a
A/duck/Bangladesh/49743/2021_H5N1
A/sonali_chicken/Bangladesh/DP−12−18/2024_H5N1_2.3.2.1a
A/mallard_duck/Bangladesh/55969/2023_H5N1_2.3.2.1a
A/duck/Bangladesh/18D2049/2023_H5N1_2.3.2.1a
A/duck/Bangladesh/49933/2021_H5N1_2.3.2.1a
A/duck/Bangladesh/49927/2021_H5N1_2.3.2.1a
A/duck/Bangladesh/35924/2018_H5N1_2.3.2.1a
A/duck/Bangladesh/38175/2019_H5N1_2.3.2.1a
A/Nepal/19FL1997/2019_H5N1_2.3.2.1a
A/goose/Bangladesh/18D1659/2021_H5N1_2.3.2.1a
A/India/SARI−4571/2021_H5N1_2.3.2.1a
A/chicken/Bangladesh/NRL−AI−2109/2017_H5N1_2.3.2.1a
A/duck/Bangladesh/30884/2016_H5N1_2.3.2.1a
A/duck/Bangladesh/17−D−2338/2024_H5N1_2.3.2.1a
A/duck/Bangladesh/17−D−2350/2024_H5N1_2.3.2.1a
A/duck/Bangladesh/58658/2023_H5N1_2.3.4.4b
A/large−billed_crow/Saitama/1101020/2023_H5N1_2.3.4.4b
A/wild_duck/Shandong/628/2011_H5N1_2.3.4.4b
A/feline/Texas/24−029329−01/2024_H5N1_2.3.4.4b
A/feline/Texas/24−029329−02/2024_H5N1_2.3.4.4b
A/dairy_cow/Texas/24_009109−002/2024_H5N1_2.3.4.4b
A/dairy_cow/South_Dakota/24_010354−011/2024_H5N1_2.3.4.4b
A/Texas/37/2024_H5N1_2.3.4.4b
A/mallard/North_Dakota/16−036222−1/2016_H5N1_Am−nonGsGD
2.3.2.1a
2.3.4.4b
0.02

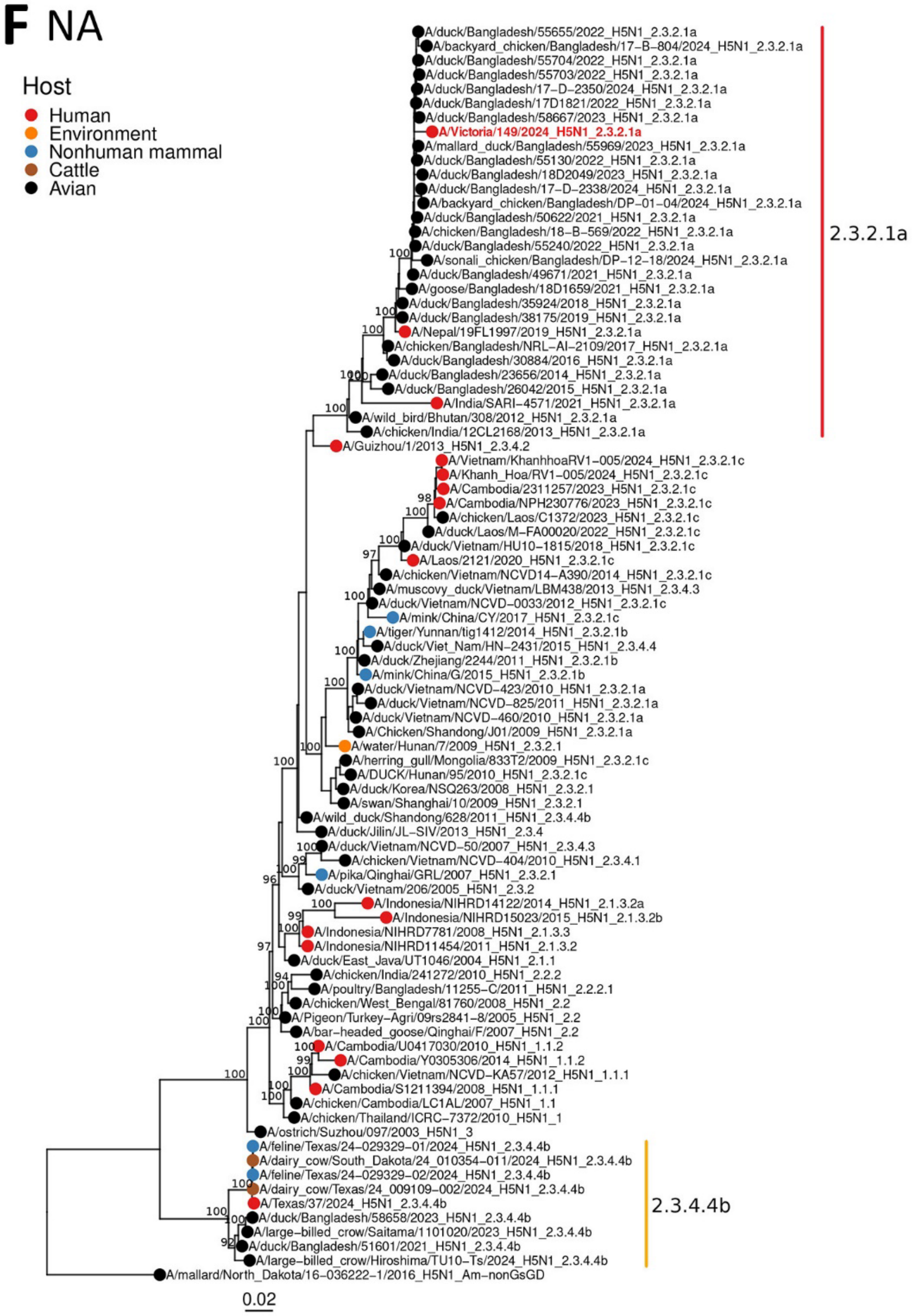

F NA
Host
Human
Environment
Nonhuman mammal
Cattle
Avian
A/duck/Bangladesh/55655/2022_H5N1_2.3.2.1a
A/backyard_chicken/Bangladesh/17-B-804/2024_H5N1_2.3.2.1a
A/duck/Bangladesh/55704/2022_H5N1_2.3.2.1a
A/duck/Bangladesh/55703/2022_H5N1_2.3.2.1a
A/duck/Bangladesh/17-D-2350/2024_H5N1_2.3.2.1a
A/duck/Bangladesh/17D1821/2022_H5N1_2.3.2.1a
A/duck/Bangladesh/58667/2023_H5N1_2.3.2.1a
A/Victoria/149/2024_H5N1_2.3.2.1a
A/mallard_duck/Bangladesh/55969/2023_H5N1_2.3.2.1a
A/duck/Bangladesh/55130/2022_H5N1_2.3.2.1a
A/duck/Bangladesh/18D2049/2023_H5N1_2.3.2.1a
A/duck/Bangladesh/17-D-2338/2024_H5N1_2.3.2.1a
A/backyard_chicken/Bangladesh/DP-01-04/2024_H5N1_2.3.2.1a
A/duck/Bangladesh/50622/2021_H5N1_2.3.2.1a
A/chicken/Bangladesh/18-B-569/2022_H5N1_2.3.2.1a
A/duck/Bangladesh/55240/2022_H5N1_2.3.2.1a
A/sonali_chicken/Bangladesh/DP-12-18/2024_H5N1_2.3.2.1a
A/duck/Bangladesh/49671/2021_H5N1_2.3.2.1a
A/goose/Bangladesh/18D1659/2021_H5N1_2.3.2.1a
A/duck/Bangladesh/35924/2018_H5N1_2.3.2.1a
A/duck/Bangladesh/38175/2019_H5N1_2.3.2.1a
A/Nepal/19FL1997/2019_H5N1_2.3.2.1a
A/chicken/Bangladesh/NRL-AI-2109/2017_H5N1_2.3.2.1a
A/duck/Bangladesh/30884/2016_H5N1_2.3.2.1a
A/duck/Bangladesh/23656/2014_H5N1_2.3.2.1a
A/duck/Bangladesh/26042/2015_H5N1_2.3.2.1a
A/India/SARI-4571/2021_H5N1_2.3.2.1a
A/wild_bird/Bhutan/308/2012_H5N1_2.3.2.1a
A/chicken/India/12CL2168/2013_H5N1_2.3.2.1a
2.3.2.1a
A/Guizhou/1/2013_H5N1_2.3.4.2
A/Vietnam/KhanhhoaRV1-005/2024_H5N1_2.3.2.1c
A/Khanh_Hoa/RV1-005/2024_H5N1_2.3.2.1c
A/Cambodia/2311257/2023_H5N1_2.3.2.1c
A/Cambodia/NPH230776/2023_H5N1_2.3.2.1c
A/chicken/Laos/C1372/2023_H5N1_2.3.2.1c
A/duck/Laos/M-FA00020/2022_H5N1_2.3.2.1c
A/duck/Vietnam/HU10-1815/2018_H5N1_2.3.2.1c
A/Laos/2121/2020_H5N1_2.3.2.1c
A/chicken/Vietnam/NCVD14-A390/2014_H5N1_2.3.2.1c
A/muscovy_duck/Vietnam/LBM438/2013_H5N1_2.3.4.3
A/duck/Vietnam/NCVD-0033/2012_H5N1_2.3.2.1c
A/mink/China/CY/2017_H5N1_2.3.2.1c
A/tiger/Yunnan/tig1412/2014_H5N1_2.3.2.1b
A/duck/Viet_Nam/HN-2431/2015_H5N1_2.3.4.4
A/duck/Zhejiang/2244/2011_H5N1_2.3.2.1b
A/mink/China/G/2015_H5N1_2.3.2.1b
A/duck/Vietnam/NCVD-423/2010_H5N1_2.3.2.1a
A/duck/Vietnam/NCVD-825/2011_H5N1_2.3.2.1a
A/duck/Vietnam/NCVD-460/2010_H5N1_2.3.2.1a
A/Chicken/Shandong/J01/2009_H5N1_2.3.2.1a
A/water/Hunan/7/2009_H5N1_2.3.2.1
A/herring_gull/Mongolia/833T2/2009_H5N1_2.3.2.1c
A/DUCK/Hunan/95/2010_H5N1_2.3.2.1c
A/duck/Korea/NSQ263/2008_H5N1_2.3.2.1
A/swan/Shanghai/10/2009_H5N1_2.3.2.1
A/wild_duck/Shandong/628/2011_H5N1_2.3.4.4b
A/duck/Jilin/JL-SIV/2013_H5N1_2.3.4
A/duck/Vietnam/NCVD-50/2007_H5N1_2.3.4.3
A/chicken/Vietnam/NCVD-404/2010_H5N1_2.3.4.1
A/pika/Qinghai/GRL/2007_H5N1_2.3.2.1
A/duck/Vietnam/206/2005_H5N1_2.3.2
A/Indonesia/NIHRD14122/2014_H5N1_2.1.3.2a
A/Indonesia/NIHRD15023/2015_H5N1_2.1.3.2b
A/Indonesia/NIHRD7781/2008_H5N1_2.1.3.3
A/Indonesia/NIHRD11454/2011_H5N1_2.1.3.2
A/duck/East_Java/UT1046/2004_H5N1_2.1.1
A/chicken/India/241272/2010_H5N1_2.2.2
A/poultry/Bangladesh/11255-C/2011_H5N1_2.2.2.1
A/chicken/West_Bengal/81760/2008_H5N1_2.2
A/Pigeon/Turkey-Agri/09rs2841-8/2005_H5N1_2.2
A/bar-headed_goose/Qinghai/F/2007_H5N1_2.2
A/Cambodia/U0417030/2010_H5N1_1.1.2
A/Cambodia/Y0305306/2014_H5N1_1.1.2
A/chicken/Vietnam/NCVD-KA57/2012_H5N1_1.1.1
A/Cambodia/S1211394/2008_H5N1_1.1.1
A/chicken/Cambodia/LC1AL/2007_H5N1_1.1
A/chicken/Thailand/ICRC-7372/2010_H5N1_1
A/ostrich/Suzhou/097/2003_H5N1_3
A/feline/Texas/24-029329-01/2024_H5N1_2.3.4.4b
A/dairy_cow/South_Dakota/24_010354-011/2024_H5N1_2.3.4.4b
A/feline/Texas/24-029329-02/2024_H5N1_2.3.4.4b
A/dairy_cow/Texas/24_009109-002/2024_H5N1_2.3.4.4b
A/Texas/37/2024_H5N1_2.3.4.4b
A/duck/Bangladesh/58658/2023_H5N1_2.3.4.4b
A/large-billed_crow/Saitama/1101020/2023_H5N1_2.3.4.4b
A/duck/Bangladesh/51601/2021_H5N1_2.3.4.4b
A/large-billed_crow/Hiroshima/TU10-Ts/2024_H5N1_2.3.4.4b
2.3.4.4b
A/mallard/North_Dakota/16-036222-1/2016_H5N1_Am-nonGsGD
0.02

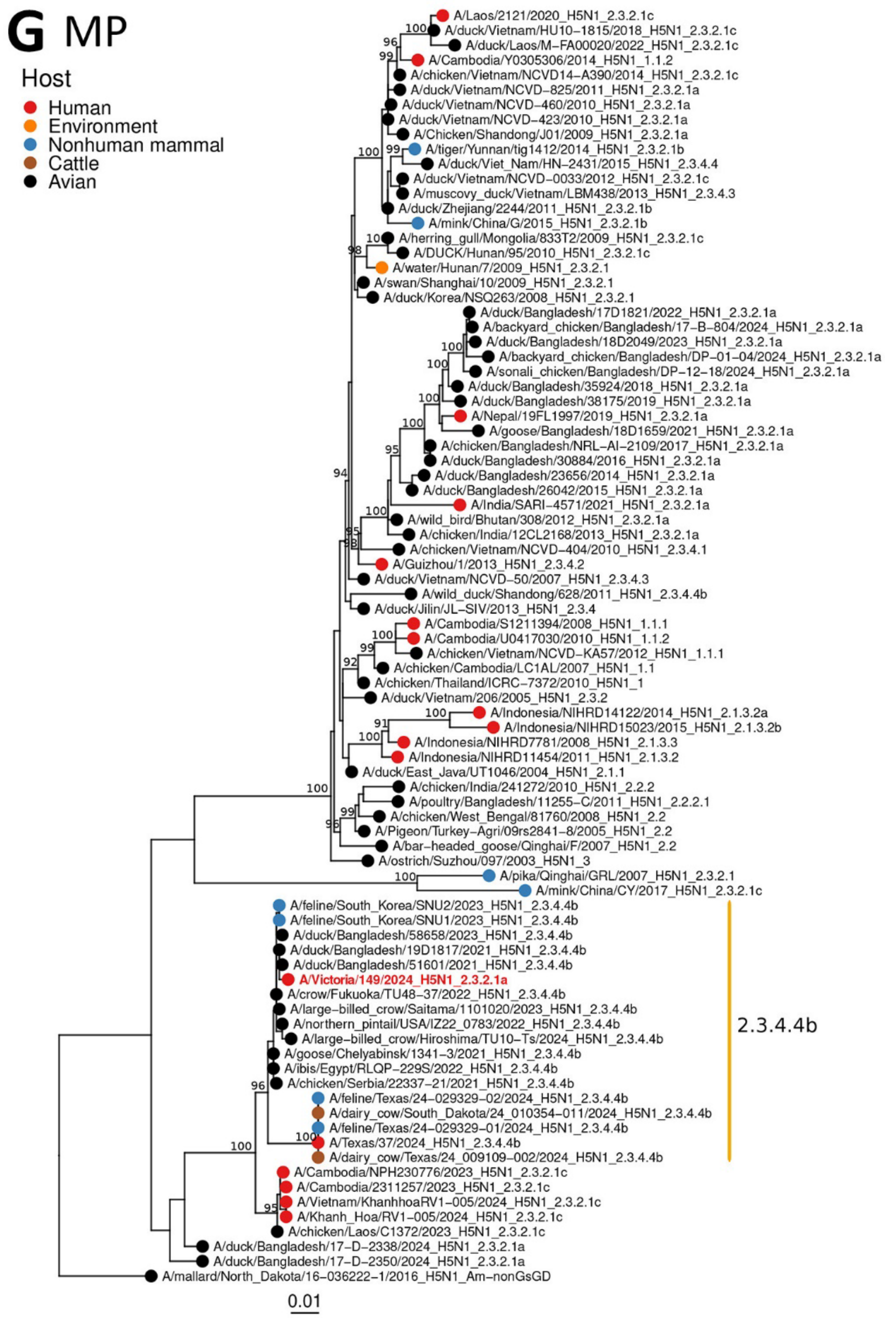
G MP
Host
Human
Environment
Nonhuman mammal
Cattle
Avian
A/Laos/2121/2020_H5N1_2.3.2.1c
A/duck/Vietnam/HU10-1815/2018_H5N1_2.3.2.1c
A/duck/Laos/M-FA00020/2022_H5N1_2.3.2.1c
A/Cambodia/Y0305306/2014_H5N1_1.1.2
A/chicken/Vietnam/NCVD14-A390/2014_H5N1_2.3.2.1c
A/duck/Vietnam/NCVD-825/2011_H5N1_2.3.2.1a
A/duck/Vietnam/NCVD-460/2010_H5N1_2.3.2.1a
A/duck/Vietnam/NCVD-423/2010_H5N1_2.3.2.1a
A/Chicken/Shandong/J01/2009_H5N1_2.3.2.1a
A/tiger/Yunnan/tig1412/2014_H5N1_2.3.2.1b
A/duck/Viet_Nam/HN-2431/2015_H5N1_2.3.4.4
A/duck/Vietnam/NCVD-0033/2012_H5N1_2.3.2.1c
A/muscovy_duck/Vietnam/LBM438/2013_H5N1_2.3.4.3
A/duck/Zhejiang/2244/2011_H5N1_2.3.2.1b
A/mink/China/G/2015_H5N1_2.3.2.1b
A/herring_gull/Mongolia/833T2/2009_H5N1_2.3.2.1c
A/DUCK/Hunan/95/2010_H5N1_2.3.2.1c
A/water/Hunan/7/2009_H5N1_2.3.2.1
A/swan/Shanghai/10/2009_H5N1_2.3.2.1
A/duck/Korea/NSQ263/2008_H5N1_2.3.2.1
A/duck/Bangladesh/17D1821/2022_H5N1_2.3.2.1a
A/backyard_chicken/Bangladesh/17-B-804/2024_H5N1_2.3.2.1a
A/duck/Bangladesh/18D2049/2023_H5N1_2.3.2.1a
A/backyard_chicken/Bangladesh/DP-01-04/2024_H5N1_2.3.2.1a
A/sonali_chicken/Bangladesh/DP-12-18/2024_H5N1_2.3.2.1a
A/duck/Bangladesh/35924/2018_H5N1_2.3.2.1a
A/duck/Bangladesh/38175/2019_H5N1_2.3.2.1a
A/Nepal/19FL1997/2019_H5N1_2.3.2.1a
A/goose/Bangladesh/18D1659/2021_H5N1_2.3.2.1a
A/chicken/Bangladesh/NRL-AI-2109/2017_H5N1_2.3.2.1a
A/duck/Bangladesh/30884/2016_H5N1_2.3.2.1a
A/duck/Bangladesh/23656/2014_H5N1_2.3.2.1a
A/duck/Bangladesh/26042/2015_H5N1_2.3.2.1a
A/India/SARI-4571/2021_H5N1_2.3.2.1a
A/wild_bird/Bhutan/308/2012_H5N1_2.3.2.1a
A/chicken/India/12CL2168/2013_H5N1_2.3.2.1a
A/chicken/Vietnam/NCVD-404/2010_H5N1_2.3.4.1
A/Guizhou/1/2013_H5N1_2.3.4.2
A/duck/Vietnam/NCVD-50/2007_H5N1_2.3.4.3
A/wild_duck/Shandong/628/2011_H5N1_2.3.4.4b
A/duck/Jilin/JL-SIV/2013_H5N1_2.3.4
A/Cambodia/S1211394/2008_H5N1_1.1.1
A/Cambodia/U0417030/2010_H5N1_1.1.2
A/chicken/Vietnam/NCVD-KA57/2012_H5N1_1.1.1
A/chicken/Cambodia/LC1AL/2007_H5N1_1.1
A/chicken/Thailand/ICRC-7372/2010_H5N1_1
A/duck/Vietnam/206/2005_H5N1_2.3.2
A/Indonesia/NIHRD14122/2014_H5N1_2.1.3.2a
A/Indonesia/NIHRD15023/2015_H5N1_2.1.3.2b
A/Indonesia/NIHRD7781/2008_H5N1_2.1.3.3
A/Indonesia/NIHRD11454/2011_H5N1_2.1.3.2
A/duck/East_Java/UT1046/2004_H5N1_2.1.1
A/chicken/India/241272/2010_H5N1_2.2.2
A/poultry/Bangladesh/11255-C/2011_H5N1_2.2.2.1
A/chicken/West_Bengal/81760/2008_H5N1_2.2
A/Pigeon/Turkey-Agri/09rs2841-8/2005_H5N1_2.2
A/bar-headed_goose/Qinghai/F/2007_H5N1_2.2
A/ostrich/Suzhou/097/2003_H5N1_3
A/pika/Qinghai/GRL/2007_H5N1_2.3.2.1
A/mink/China/CY/2017_H5N1_2.3.2.1c
A/feline/South_Korea/SNU2/2023_H5N1_2.3.4.4b
A/feline/South_Korea/SNU1/2023_H5N1_2.3.4.4b
A/duck/Bangladesh/58658/2023_H5N1_2.3.4.4b
A/duck/Bangladesh/19D1817/2021_H5N1_2.3.4.4b
A/duck/Bangladesh/51601/2021_H5N1_2.3.4.4b
A/Victoria/149/2024_H5N1_2.3.2.1a
A/crow/Fukuoka/TU48-37/2022_H5N1_2.3.4.4b
A/large-billed_crow/Saitama/1101020/2023_H5N1_2.3.4.4b
A/northern_pintail/USA/IZ22_0783/2022_H5N1_2.3.4.4b
A/large-billed_crow/Hiroshima/TU10-Ts/2024_H5N1_2.3.4.4b
A/goose/Chelyabinsk/1341-3/2021_H5N1_2.3.4.4b
A/ibis/Egypt/RLQP-229S/2022_H5N1_2.3.4.4b
A/chicken/Serbia/22337-21/2021_H5N1_2.3.4.4b
A/feline/Texas/24-029329-02/2024_H5N1_2.3.4.4b
A/dairy_cow/South_Dakota/24_010354-011/2024_H5N1_2.3.4.4b
A/feline/Texas/24-029329-01/2024_H5N1_2.3.4.4b
A/Texas/37/2024_H5N1_2.3.4.4b
A/dairy_cow/Texas/24_009109-002/2024_H5N1_2.3.4.4b
A/Cambodia/NPH230776/2023_H5N1_2.3.2.1c
A/Cambodia/2311257/2023_H5N1_2.3.2.1c
A/Vietnam/KhanhhoaRV1-005/2024_H5N1_2.3.2.1c
A/Khanh_Hoa/RV1-005/2024_H5N1_2.3.2.1c
A/chicken/Laos/C1372/2023_H5N1_2.3.2.1c
A/duck/Bangladesh/17-D-2338/2024_H5N1_2.3.2.1a
A/duck/Bangladesh/17-D-2350/2024_H5N1_2.3.2.1a
A/mallard/North_Dakota/16-036222-1/2016_H5N1_Am-nonGsGD
2.3.4.4b
0.01

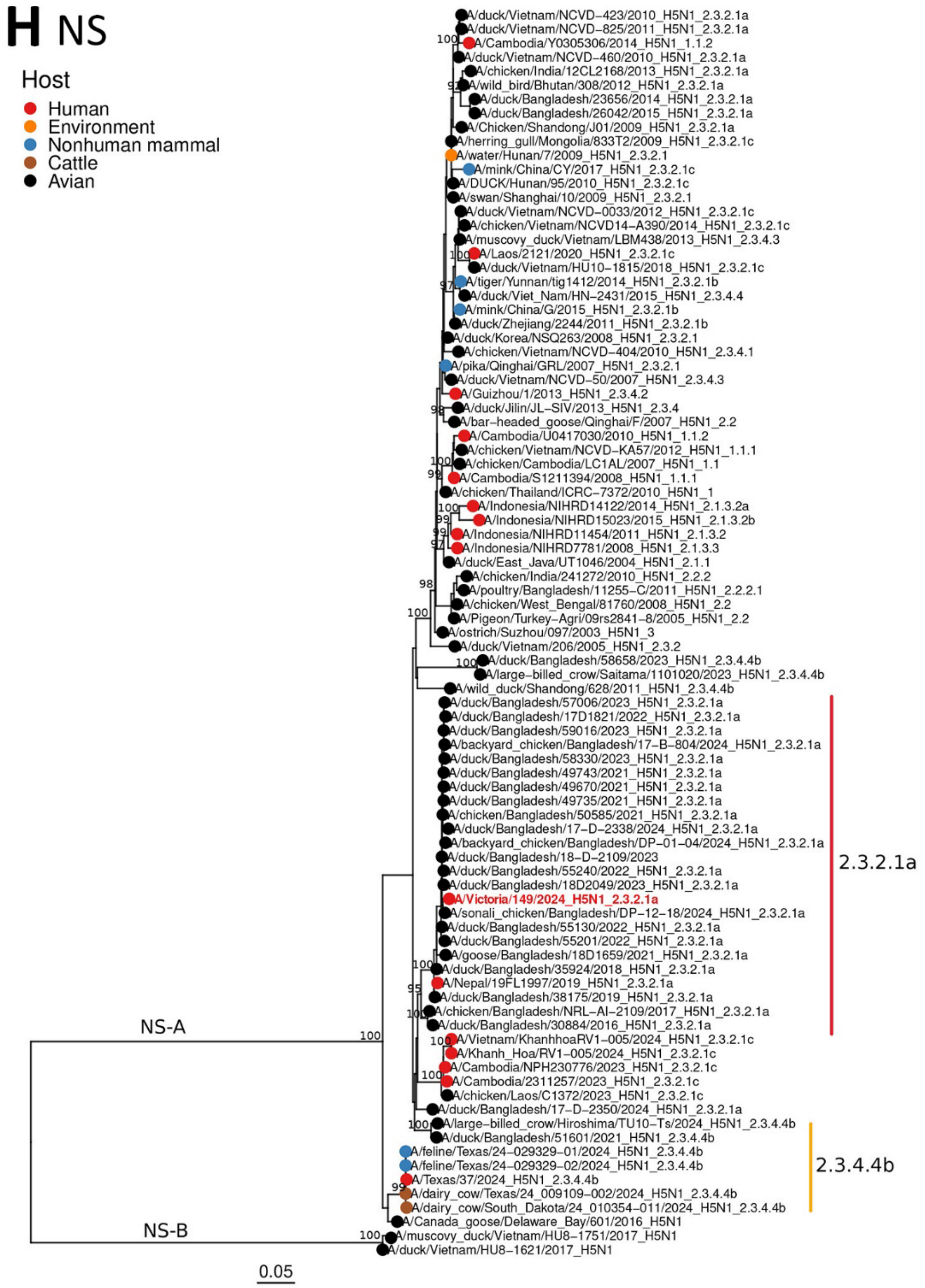
H NS
Host
Human
Environment
Nonhuman mammal
Cattle
Avian
A/duck/Vietnam/NCVD-423/2010_H5N1_2.3.2.1a
A/duck/Vietnam/NCVD-825/2011_H5N1_2.3.2.1a
A/Cambodia/Y0305306/2014_H5N1_1.1.2
A/duck/Vietnam/NCVD-460/2010_H5N1_2.3.2.1a
A/chicken/India/12CL2168/2013_H5N1_2.3.2.1a
A/wild_bird/Bhutan/308/2012_H5N1_2.3.2.1a
A/duck/Bangladesh/23656/2014_H5N1_2.3.2.1a
A/duck/Bangladesh/26042/2015_H5N1_2.3.2.1a
A/Chicken/Shandong/J01/2009_H5N1_2.3.2.1a
A/herring_gull/Mongolia/833T2/2009_H5N1_2.3.2.1c
A/water/Hunan/7/2009_H5N1_2.3.2.1
A/mink/China/CY/2017_H5N1_2.3.2.1c
A/DUCK/Hunan/95/2010_H5N1_2.3.2.1c
A/swan/Shanghai/10/2009_H5N1_2.3.2.1
A/duck/Vietnam/NCVD-0033/2012_H5N1_2.3.2.1c
A/chicken/Vietnam/NCVD14-A390/2014_H5N1_2.3.2.1c
A/muscovy_duck/Vietnam/LBM438/2013_H5N1_2.3.4.3
A/Laos/2121/2020_H5N1_2.3.2.1c
A/duck/Vietnam/HU10-1815/2018_H5N1_2.3.2.1c
A/tiger/Yunnan/tig1412/2014_H5N1_2.3.2.1b
A/duck/Viet_Nam/HN-2431/2015_H5N1_2.3.4.4
A/mink/China/G/2015_H5N1_2.3.2.1b
A/duck/Zhejiang/2244/2011_H5N1_2.3.2.1b
A/duck/Korea/NSQ263/2008_H5N1_2.3.2.1
A/chicken/Vietnam/NCVD-404/2010_H5N1_2.3.4.1
A/pika/Qinghai/GRL/2007_H5N1_2.3.2.1
A/duck/Vietnam/NCVD-50/2007_H5N1_2.3.4.3
A/Guizhou/1/2013_H5N1_2.3.4.2
A/duck/Jilin/JL-SIV/2013_H5N1_2.3.4
A/bar-headed_goose/Qinghai/F/2007_H5N1_2.2
A/Cambodia/U0417030/2010_H5N1_1.1.2
A/chicken/Vietnam/NCVD-KA57/2012_H5N1_1.1.1
A/chicken/Cambodia/LC1AL/2007_H5N1_1.1
A/Cambodia/S1211394/2008_H5N1_1.1.1
A/chicken/Thailand/ICRC-7372/2010_H5N1_1
A/Indonesia/NIHRD14122/2014_H5N1_2.1.3.2a
A/Indonesia/NIHRD15023/2015_H5N1_2.1.3.2b
A/Indonesia/NIHRD11454/2011_H5N1_2.1.3.2
A/Indonesia/NIHRD7781/2008_H5N1_2.1.3.3
A/duck/East_Java/UT1046/2004_H5N1_2.1.1
A/chicken/India/241272/2010_H5N1_2.2.2
A/poultry/Bangladesh/11255-C/2011_H5N1_2.2.2.1
A/chicken/West_Bengal/81760/2008_H5N1_2.2
A/Pigeon/Turkey-Agri/09rs2841-8/2005_H5N1_2.2
A/ostrich/Suzhou/097/2003_H5N1_3
A/duck/Vietnam/206/2005_H5N1_2.3.2
A/duck/Bangladesh/58658/2023_H5N1_2.3.4.4b
A/large-billed_crow/Saitama/1101020/2023_H5N1_2.3.4.4b
A/wild_duck/Shandong/628/2011_H5N1_2.3.4.4b
A/duck/Bangladesh/57006/2023_H5N1_2.3.2.1a
A/duck/Bangladesh/17D1821/2022_H5N1_2.3.2.1a
A/duck/Bangladesh/59016/2023_H5N1_2.3.2.1a
A/backyard_chicken/Bangladesh/17-B-804/2024_H5N1_2.3.2.1a
A/duck/Bangladesh/58330/2023_H5N1_2.3.2.1a
A/duck/Bangladesh/49743/2021_H5N1_2.3.2.1a
A/duck/Bangladesh/49670/2021_H5N1_2.3.2.1a
A/duck/Bangladesh/49735/2021_H5N1_2.3.2.1a
A/chicken/Bangladesh/50585/2021_H5N1_2.3.2.1a
A/duck/Bangladesh/17-D-2338/2024_H5N1_2.3.2.1a
A/backyard_chicken/Bangladesh/DP-01-04/2024_H5N1_2.3.2.1a
A/duck/Bangladesh/18-D-2109/2023
A/duck/Bangladesh/55240/2022_H5N1_2.3.2.1a
A/duck/Bangladesh/18D2049/2023_H5N1_2.3.2.1a
A/Victoria/149/2024_H5N1_2.3.2.1a
A/sonali_chicken/Bangladesh/DP-12-18/2024_H5N1_2.3.2.1a
A/duck/Bangladesh/55130/2022_H5N1_2.3.2.1a
A/duck/Bangladesh/55201/2022_H5N1_2.3.2.1a
A/goose/Bangladesh/18D1659/2021_H5N1_2.3.2.1a
A/duck/Bangladesh/35924/2018_H5N1_2.3.2.1a
A/Nepal/19FL1997/2019_H5N1_2.3.2.1a
A/duck/Bangladesh/38175/2019_H5N1_2.3.2.1a
A/chicken/Bangladesh/NRL-AI-2109/2017_H5N1_2.3.2.1a
A/duck/Bangladesh/30884/2016_H5N1_2.3.2.1a
A/Vietnam/KhanhhoaRV1-005/2024_H5N1_2.3.2.1c
A/Khanh_Hoa/RV1-005/2024_H5N1_2.3.2.1c
A/Cambodia/NPH230776/2023_H5N1_2.3.2.1c
A/Cambodia/2311257/2023_H5N1_2.3.2.1c
A/chicken/Laos/C1372/2023_H5N1_2.3.2.1c
A/duck/Bangladesh/17-D-2350/2024_H5N1_2.3.2.1a
A/large-billed_crow/Hiroshima/TU10-Ts/2024_H5N1_2.3.4.4b
A/duck/Bangladesh/51601/2021_H5N1_2.3.4.4b
A/feline/Texas/24-029329-01/2024_H5N1_2.3.4.4b
A/feline/Texas/24-029329-02/2024_H5N1_2.3.4.4b
A/Texas/37/2024_H5N1_2.3.4.4b
A/dairy_cow/Texas/24_009109-002/2024_H5N1_2.3.4.4b
A/dairy_cow/South_Dakota/24_010354-011/2024_H5N1_2.3.4.4b
A/Canada_goose/Delaware_Bay/601/2016_H5N1
A/muscovy_duck/Vietnam/HU8-1751/2017_H5N1
A/duck/Vietnam/HU8-1621/2017_H5N1
2.3.2.1a
2.3.4.4b
NS-A
NS-B
0.05

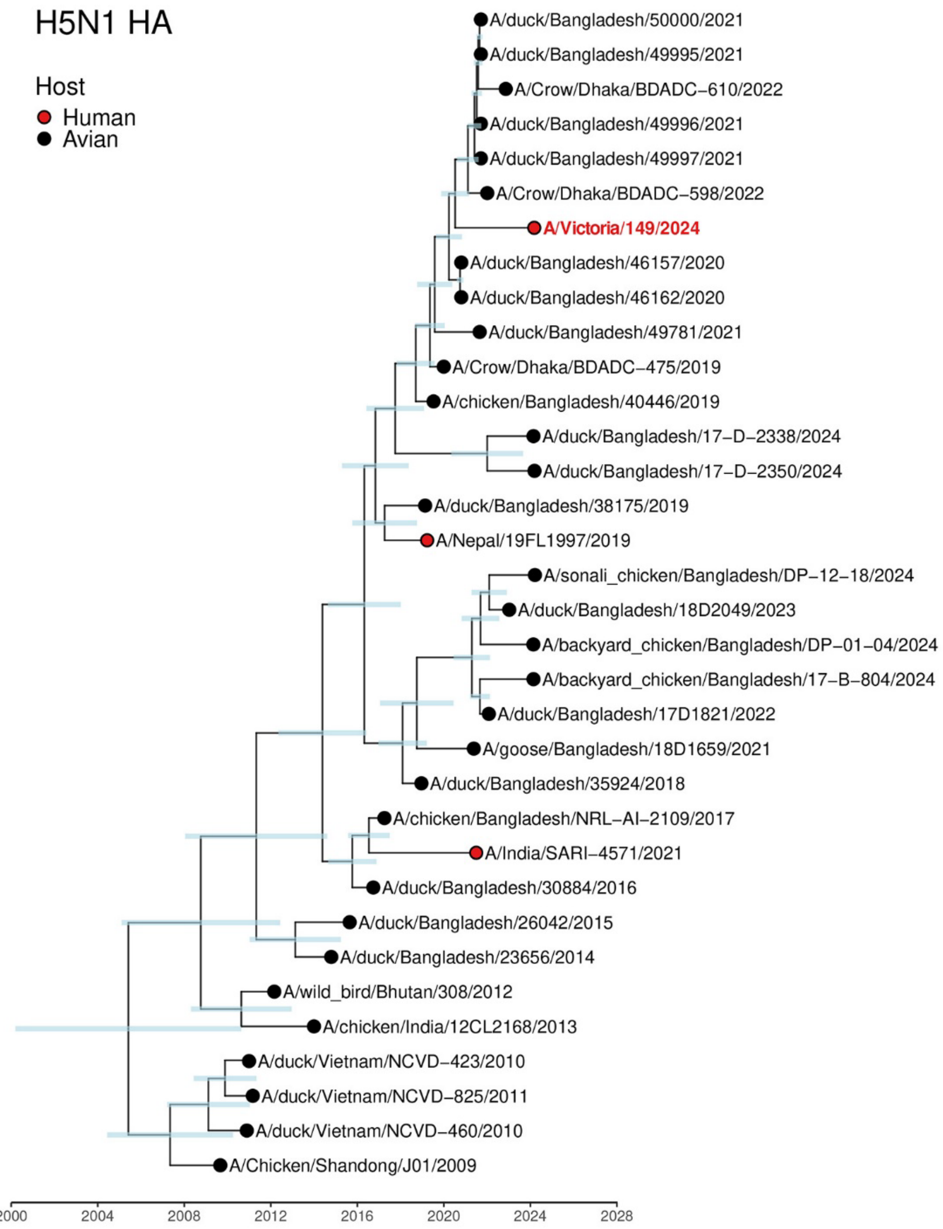

Appendix Figure 2. A time structured phylogenetic analysis of the H5N1 clade 2.3.2.1a viruses shown in Figure 1 (main article). See methods described previously in Appendix.
